# Stimulation of the *Caulobacter crescentus* surface sensing pathway by deletion of a specialized minor pilin-like gene

**DOI:** 10.1128/mbio.02302-25

**Published:** 2025-10-01

**Authors:** Farah Obeid Charrouf, Gregory B. Whitfield, Courtney K. Ellison, Yves V. Brun

**Affiliations:** 1 Département de Microbiologie, Infectiologie et Immunologie, Université de Montréal 5622, Montreal, Québec, Canada; 2 Department of Microbiology, University of Georgia 189270, Athens, Georgia, USA; Albert-Ludwigs-Universitat Freiburg, Freiburg, Germany

**Keywords:** surface-sensing, pili, biofilms

## Abstract

Bacteria colonize surfaces through complex mechanisms of surface sensing. Pili are dynamic bacterial appendages that play an important role in this process. In *Caulobacter crescentus*, the tension on retracting, surface-bound pili triggers the rapid synthesis of holdfast, an adhesive that permanently attaches cells to surfaces. However, the mechanosensory elements in pilus-mediated surface sensing remain unknown. In this study, we used a genetic screen to isolate mutants with altered pilus dynamics, to identify genes potentially involved in pilus-mediated surface sensing. This screen identified *cpaL*, whose deletion led to reduced piliation levels, but surprisingly also to increased holdfast production and attachment—an unexpected finding given the importance of pili in these processes. Interestingly, this phenotype was absent under conditions that restrict the surface-sensing pathway of holdfast production. Furthermore, when pilus retraction was blocked to artificially simulate surface contact, ∆*cpaL* cells did not show increased holdfast production as seen in wild-type cells. Together, these observations suggest that loss of *cpaL* stimulates surface sensing even in the absence of mechanical cues, consistent with a negative regulatory role for CpaL in the surface-sensing pathway of holdfast production in *C. crescentus*. Structural predictions suggest that CpaL is a minor pilin fused to a mechanosensitive von Willebrand factor type A (vWA) domain, which can be accommodated at the pilus tip alongside other minor pilins. These results collectively position CpaL as a strong candidate for the mechanosensory element in pilus-mediated surface sensing in *C. crescentus*.

**IMPORTANCE:**

Surface sensing allows bacteria to colonize surfaces and form biofilms, with wide-ranging implications for bacterial survival, ecology, and human health. In *Caulobacter crescentus*, tight adherence (Tad) pili play an important role in surface sensing and attachment, however the molecular mechanisms of pilus-mediated mechanosensing remain unknown. Here, we demonstrate that CpaL, a potential pilus tip mechanosensory protein, could be a major regulatory element controlling Tad pilus-mediated surface attachment and colonization in *C. crescentus*. Specifically, CpaL plays a regulatory role in holdfast synthesis upon surface contact. By identifying CpaL as a key player in surface recognition, our work offers valuable insights into the mechanisms of bacterial adhesion.

## INTRODUCTION

Surface recognition and attachment are essential steps in the process of bacterial surface colonization and biofilm formation (1, 2). To sense surfaces, bacteria respond to physical or mechanical cues, which trigger phenotypic changes that transform free-swimming bacterial cells into surface-adherent cells that form biofilms (3–5). The process by which bacterial cells sense surfaces and convert this signal into downstream cellular processes is called surface sensing (6, 7). A great deal of effort has been expended in deciphering the mechanisms of surface sensing in bacteria, establishing a pivotal role for extracellular appendages, such as flagella and pili, in this process (8–11).

Pili are thread-like, proteinaceous appendages that physically interact with their surroundings and are critical for many important cellular processes, such as adherence, aggregation, biofilm formation, horizontal gene transfer, and virulence in some pathogenic species (12, 13). Pili are composed of thousands of repeats of a small protein subunit, the major pilin, and less abundant minor pilin(s) (12, 14). A subset of pili, called the type IV pili (T4P), are characterized by their dynamic activity, exhibiting the ability to extend away from the cell surface and subsequently retract back into the cell by polymerization and depolymerization of the pilus fiber, respectively. This is achieved by a complex membrane-spanning machine that draws from a pool of pilins in the inner membrane to assemble the fiber, which passes through an outer membrane channel (14). Based primarily on differences in the motor components of the T4P machinery, recent phylogenetic analyses have divided the T4P into three subclasses: T4aP, T4bP, and T4cP (11, 15, 16). Among these, T4cP, also known as tight adherence (Tad) pili, are thought to have evolved from an archaeal ancestor. Tad pili are broadly distributed among bacteria, including in the freshwater bacterium *Caulobacter crescentus*, where they have been implicated in adhesion and surface sensing (11, 17).

*C. crescentus* exhibits a dimorphic life cycle, wherein each cell division produces a non-motile stalked cell and a motile swarmer cell harboring multiple Tad pili and one flagellum at the same pole (18). The swarmer cell subsequently undergoes differentiation as it developmentally progresses through the cell cycle, whereupon it synthesizes a compositionally complex adhesin called holdfast to mediate permanent surface attachment, followed by stalk formation at the holdfast-bearing pole (10, 18, 19). Interestingly, surface contact by a swarmer cell can hasten differentiation by rapidly triggering multiple processes, starting with the retraction of the pili into the cell, cessation of further dynamic activity by the pili, ejection of the flagellum, and finally synthesis of the holdfast (11). While *C. crescentus* can synthesize holdfast through the cell-cycle-mediated developmental pathway even in the absence of pili or a surface, the surface-contact-mediated production of holdfast requires the presence of pili, and physical obstruction of pilus retraction leads to rapid holdfast synthesis even in the absence of surface contact, implying that tension on retracting, surface-bound pili may be a cue to sense surface contact (11, 20). However, the mechanistic details of how Tad pili sense surfaces, convey the signal across the cell envelope, and translate it into an output capable of upregulating surface-associated behaviors, remain poorly understood.

In this study, we address these knowledge gaps in our mechanistic understanding of Tad pilus-mediated mechanosensing. We performed a genetic screen that identified mutations in *cpaL*, whose deletion led to significantly reduced piliation levels. However, despite reduced pilus synthesis, we found that this mutant showed increased surface adherence and holdfast synthesis. Stimulation of holdfast production was not observed when the *cpaL* mutant was cultured in a defined medium that nutritionally restricts surface sensing. In addition, holdfast synthesis did not increase when pilus retraction was blocked in the *cpaL* mutant to stimulate surface sensing. Finally, we examined the predicted structure of CpaL using AlphaFold3 and found that CpaL is comprised of a pilin-like module connected to a von Willebrand factor type A (vWA) domain, a fold that is often implicated in mechanosensing. Further modelling suggests that CpaL could form a complex with the minor pilins CpaJ and CpaK, which can be accommodated at the distal end of the pilus. Collectively, these data suggest that CpaL plays an important role in the pilus-mediated surface sensing pathway of *C. crescentus*.

## RESULTS

### A screen for mutations conferring resistance to the pilus-dependent phage φCbK identifies the *cpaL* gene

Tad pili exhibit dynamic cycles of extension and retraction, which play a crucial role in surface-sensing in *C. crescentus* (11). To identify genes potentially involved in pilus dynamics, in particular pilus retraction, we performed a genetic screen to identify mutants with some degree of piliation, which were nonetheless resistant to the pilus-dependent phage φCbK. Previous studies have shown that once φCbK attaches to *Caulobacter* pili outside the cell, pilus retraction is essential to facilitate phage adsorption into the cell (21, 22). Thus, we reasoned that mutations that impair pilus retraction would increase resistance to this phage. To carry out our screen and all subsequent analyses, we used *C. crescentus pilA-cys* as the parent background, where WT cells are modified to incorporate a cysteine residue in the major pilin PilA, allowing for pilus labeling and modulation (11, 23). A pooled transposon mutant library was generated in the parent strain and then mixed with φCbK, and phage resistant mutants were isolated (Fig. S1). The ability of these mutants to elaborate a pilus was analyzed via microscopy by labeling pili with the thiol-reactive fluorophore AF488-maleimide (11, 23). A total of 184 mutants were identified that conferred resistance to φCbK. After labeling pili, these mutants fell into four categories: (i) cells that exhibited no pilus labeling, (ii) cells with aberrant growth that may or may not have exhibited pilus labeling, (iii) cells with fluorescently labeled cell bodies but no visible pili, and (iv) cells with fluorescently labeled pilus filaments. Of these, categories 2–4 were considered of interest for the identification of mutants with potential defects in pilus retraction. This resulted in further characterization of 25 mutants with complete or partial phage resistance along with variable pilus fluorescence profiles, which were sequenced to identify the sites of transposon insertion (Table S1). Among these, the gene with the most transposon insertions was *cpaL* (*CCNA_0199*), with four hits (Fig. S1). *cpaL* is an orphan gene located outside the main pilus gene cluster, and has previously been shown to impact phage φCbK propagation (24).

**Fig 1:**
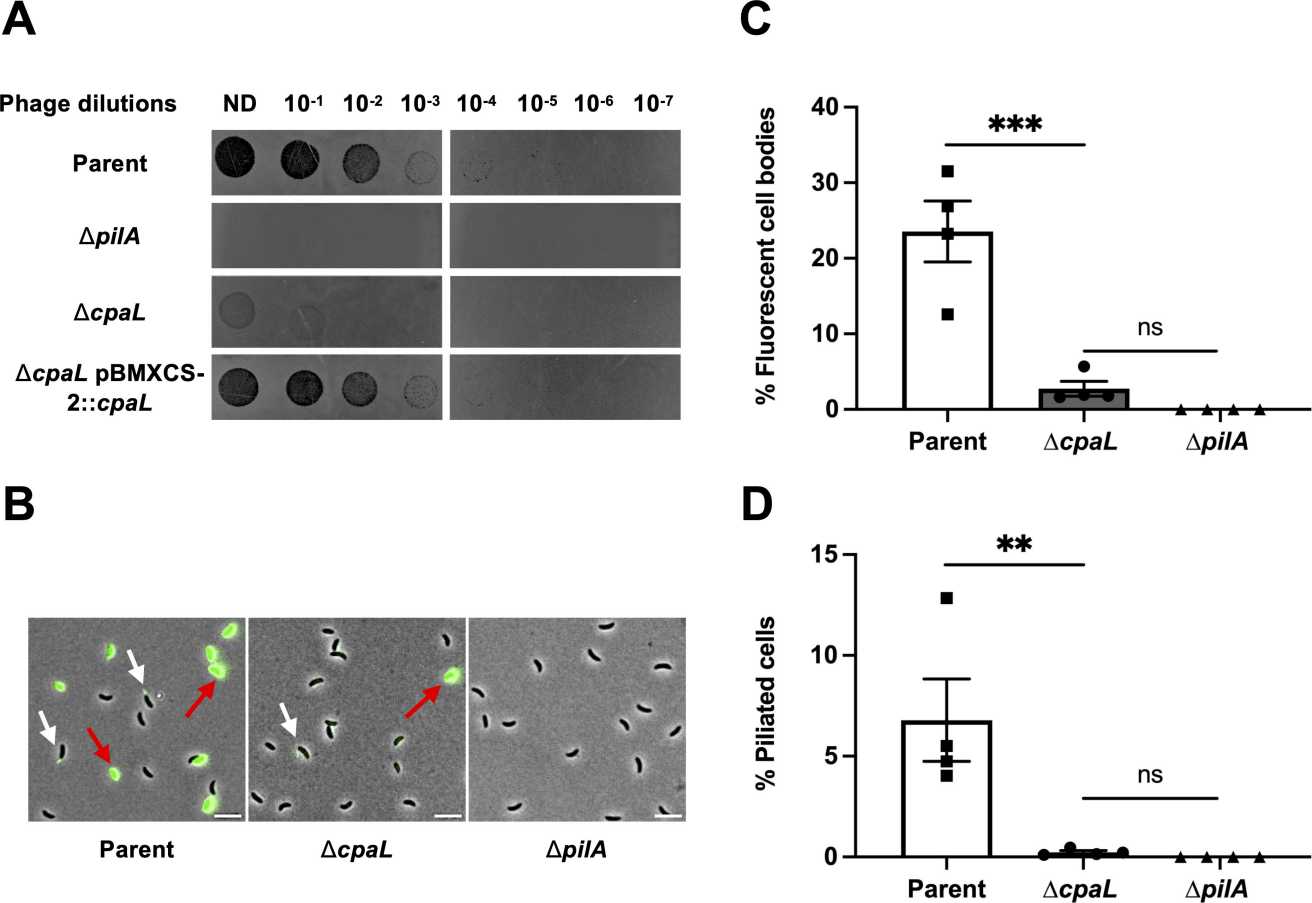
Deletion of *cpaL* reduces the sensitivity of *C. crescentus* to the pilus-dependent phage ΦCbK. (**A**) Top agar phage sensitivity assays for the parent strain (NA1000 *pilA*-cys) and the isogenic mutants Δ*pilA* (phage-resistant strain lacking pili) and Δ*cpaL*, each carrying empty plasmid vectors, as well as the Δ*cpaL* strain carrying a plasmid-borne copy of *cpaL* (pBMXCS-2::*cpaL*). Serial dilutions of the phage were spotted onto plates with top agar containing each bacterial strain. ND, no dilution. (**B**) Representative microscopy images for synchronized swarmer cells of the parent strain and the isogenic Δ*cpaL* mutant, labeled with the AF488-maleimide dye (green), which reacts with the engineered cysteine residue in the major pilin, PilA. Scale bars, 10 µm. White arrows indicate cells with labeled pili, red arrows indicate fluorescent cell bodies. (**C**) Quantification of the percentage of cells with fluorescent cell bodies in synchronized populations of the indicated strains labeled with AF488-maleimide. (**D**) Quantification of the percentage of piliated cells in synchronized swarmer cells of the indicated strains labeled with AF488-maleimide dye (green). Results are the mean of four independent biological replicates, with at least 500 cells analyzed per replicate. Error bars represent the standard error of the mean (SEM). Statistical comparisons were made using Tukey’s multiple comparisons test. ***, *P* < 0.001; **, *P* < 0.01; ns, no significant difference.

We generated an unmarked, in-frame deletion of *cpaL* and assessed the sensitivity of this mutant to ΦCbK. We spotted serial dilutions of φCbK onto growth plates with *C. crescentus* incorporated into the top agar and analyzed the formation of plaques due to phage infection (Fig. 1A). The phage-susceptible parent strain exhibited the formation of clear plaques up to the 10^−5^ phage dilution, while the pilus-deficient mutant Δ*pilA* displayed complete resistance to φCbK. In contrast, the Δ*cpaL* mutant demonstrated intermediate resistance to φCbK, with formation of cloudy plaques visible only up to the 10^−1^ phage dilution. The φCbK resistance phenotype in Δ*cpaL* was completely reversed when complemented with a wild-type copy of *cpaL* on a replicating plasmid (Fig. 1A). The growth curves of all the analyzed strains were comparable, indicating that φCbK resistance in the Δ*cpaL* mutant is not due to a variation in growth rate (Fig. S2). Together, these results implicate *cpaL* in pilus-related processes affecting phage susceptibility, which motivated further investigation into its involvement in pilus synthesis and/or dynamics.

### The Δ*cpaL* mutant produces fewer pili per cell, but with an increased average length

Since the Δ*cpaL* mutant was less sensitive to the pilus-dependent phage φCbK, we hypothesized that this could be due to a decrease in the amount of pilus retraction within the population. To visualize pilus retraction, we quantified cells with fluorescent cell bodies in *C. crescentus* (Fig. 1B), since cell body fluorescence results from the retraction of labeled pilins into the cell (11). We quantified the proportion of fluorescent cell bodies among synchronized swarmer cells stained for pili, comparing the parent and mutant strains. Approximately 23.5% of synchronized swarmer cells of the parent strain had fluorescent cell bodies after labeling, consistent with previous reports (11, 25). In contrast, less than 3% of the Δ*cpaL* synchronized swarmer cells displayed fluorescent cell bodies (Fig. 1B and C). Moreover, approximately 6% of parent cells had visible pili, whereas piliated cells were rarely observed for the Δ*cpaL* mutant (Fig. 1B and D). The ∆*pilA* mutant lacking pili served as a negative control, showing no fluorescence. Together, these results indicate that Δ*cpaL* cells produce significantly fewer pili than their parental strain, but that those pili that are produced can retract. The dynamic activity of pili produced by the Δ*cpaL* mutant was examined by time-lapse microscopy, which revealed a distribution of dynamic behaviors that is consistent with what has been observed previously for *C. crescentus* (Movies S1 and S2) (11).

**Fig 2:**
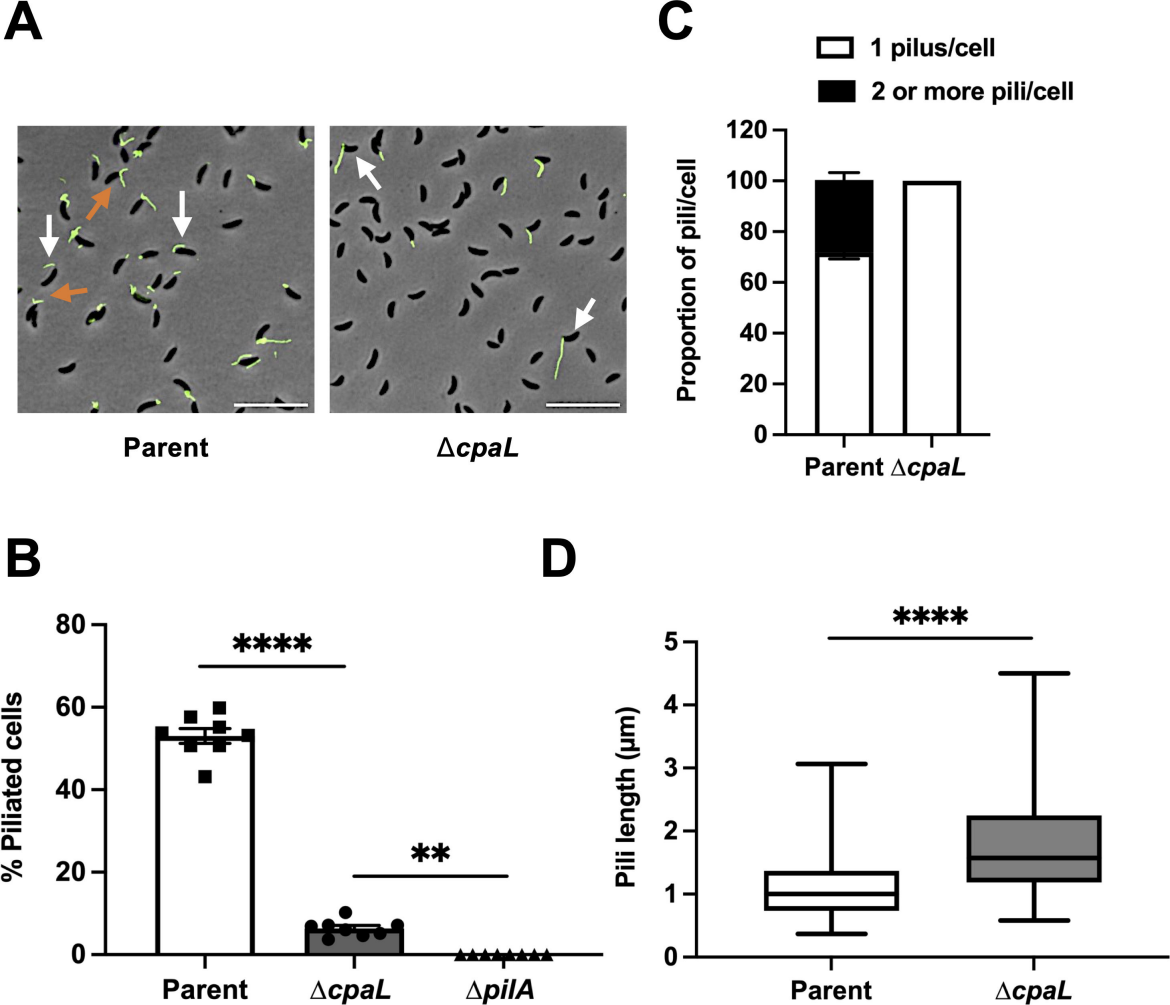
Deletion of *cpaL* reduces piliation, but increases the average length of the pilus fiber. (**A**) Representative microscopy images of synchronized swarmer cells of the parent strain (NA1000 *pilA*-cys) and the isogenic Δ*cpaL* mutant, whose pili are labeled with AF488-maleimide (green) after blocking pilus retraction using PEG5000-mal, which reacts with the engineered cysteine residue in PilA. White arrows indicate cells with a single pilus per cell. Orange arrows indicate cells with two or more pili per cell. Scale bars, 10 µm. (**B**) Quantification of the percentage of piliated cells in synchronized populations of the indicated strains, when pilus retraction was blocked with PEG5000-mal. Results are the mean of eight independent biological replicates, with at least 200 cells analyzed per replicate. Error bars represent the standard error of the mean. Statistical comparisons were made using Tukey’s multiple comparisons test. (**C**) Quantification of the number of pili produced per piliated cell of the indicated strains, measured in synchronized populations after blocking pilus retraction with PEG5000-mal. Results are the mean of four independent biological replicates with at least 200 cells analyzed per replicate. Error bars represent the standard error of the mean. (**D**) Average length of pili produced by synchronized swarmer cells of the indicated strains after blocking pilus retraction with PEG5000-mal. One hundred pili were measured for each strain. Error bars indicate the minimum to maximum range of lengths. Statistical comparison was made using a two-tailed unpaired *t*-test. ****, *P* < 0.0001; **, *P* < 0.01.

Next, to visualize the overall levels of pilus synthesis by these strains, we incubated synchronized parental and Δ*cpaL* swarmer cells with PEG5000-maleimide (PEG5000-mal) to block pilus retraction, through its reaction with the modified PilA cysteine residue, while simultaneously labeling pili before imaging, as described previously (11). We found that around 53% of the parental swarmer cell population exhibited piliation (Fig. 2A and B). In contrast, around 6% of swarmer cells from the Δ*cpaL* mutant population produced pili (Fig. 2A and B). Finally, the production of pili was restored to parental levels when the Δ*cpaL* mutant was complemented with plasmid-borne wild-type *cpaL* (Fig. S3A).

We further analyzed the number of pili produced by each piliated cell under these conditions (with blocked pilus retraction) and found that the Δ*cpaL* mutant produced only one pilus per piliated cell, in contrast to the parent strain, where 29% of piliated cells elaborated more than one pilus (Fig. 2A and C). In addition, the single pilus produced by the Δ*cpaL* mutant had an average length of 1.81 μm, which is significantly longer than the pili produced by the parent strain (1.02 μm; consistent with previous reports) (11) (Fig. 2A and D). These findings suggest that CpaL may play a role in Tad pilus biosynthesis in *C. crescentus,* perhaps contributing to the initiation or regulation of pilus formation.

### Deletion of *cpaL* increases surface adhesion and holdfast production

**Fig 3:**
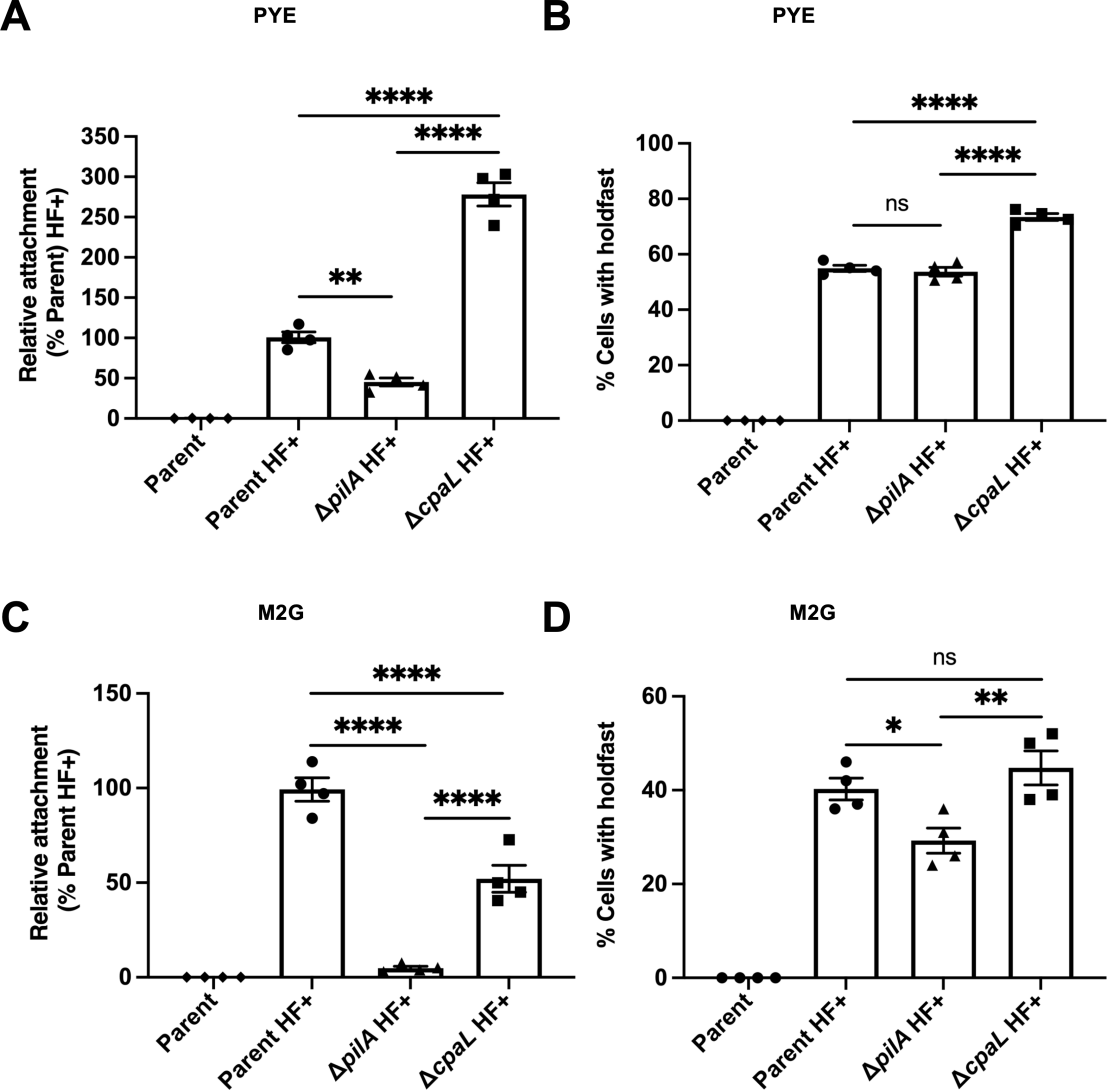
Deletion of *cpaL* increases surface attachment and holdfast production in peptone-yeast extract (PYE) but not in M2G medium. (**A**) Quantification of the attachment of cells to a glass coverslip after 30 min of incubation, when grown in the complex medium PYE. Attachment values are presented relative to the parent HF+ strain, derived from the parental NA1000 *pilA*-cys strain after repair of *hfsA* to allow for holdfast production (HF+: *hfsA+*, holdfast positive). Attachment in the isogenic ∆*pilA* and ∆*cpaL* strains carrying the HF+ modification is presented alongside the unmodified parent strain NA1000 *pilA*-cys, which lacks holdfast and serves as a negative control for attachment. (**B**) Quantification of the percentage of cells producing holdfast in the population, when grown in the complex medium PYE on an agarose pad. (**C**) Quantification of the attachment of cells to a glass coverslip after 30 min of incubation, when grown in the minimal medium M2G. Attachment values for the different strains are presented relative to the parent HF+ strain as described in panel **A**. (**D**) Quantification of the percentage of cells producing holdfast in the population, when grown in the minimal medium M2G on an agarose pad. Data are the mean of four independent biological replicates. Error bars indicate the SEM. Statistical comparisons were made using Tukey’s multiple comparisons test. ****, *P* < 0.0001; **, *P* < 0.01; *, *P* < 0.05.

It has previously been demonstrated that pili play a crucial role in surface-sensing and adhesion in *C. crescentus* by rapidly stimulating synthesis of the holdfast upon surface contact (11, 20). Therefore, we expected that the Δ*cpaL* mutant would exhibit a surface attachment defect due to its reduced piliation. To test this hypothesis, we used a surface attachment assay (20) wherein cells were spotted onto a glass coverslip and incubated for 30 min to allow attachment to the surface. The coverslip was then washed to remove unattached cells, and the surface-adherent cells were imaged by microscopy. As the parental strain is derived from NA1000, which is unable to produce a holdfast due to a point mutation in the holdfast biosynthetic gene *hfsA* (Fig. 3A; Fig. S4) (26), we repaired this mutation to generate holdfast-positive (HF+) strains in the parental background, which are amenable to surface attachment assays. The Δ*pilA* HF+ mutant exhibits around a 54% reduction in surface binding compared to the parent HF+ strain (Fig. 3A; Fig. S4). In contrast, the ∆*cpaL* HF+ mutant exhibited a three-fold increase in surface binding compared to the parent HF+ strain (Fig. 3A; Fig. S4), which is unexpected for a mutant with reduced piliation levels.

To determine whether the elevated surface adherence of Δ*cpaL* HF+ was due to increased holdfast production, we imaged holdfasts on agarose pads using a fluorescently labeled wheat-germ agglutinin lectin (AF488-WGA) that binds specifically to the *N*-acetyl-glucosamine moiety present in holdfasts (11, 27). We imaged cells on soft peptone-yeast extract (PYE) agarose pads since they minimize the stimulation of holdfast production via the surface-sensing pathway (19, 28). Holdfasts were detected in 55% of cells in the Δ*pilA* HF+ mutant and in the parent HF+ strain (Fig. 3B). In contrast, 74% of cells in the Δ*cpaL* HF+ mutant population had a holdfast (Fig. 3B). Surface binding and holdfast production were restored to the levels of the parent HF+ strain when the Δ*cpaL* HF+ mutant was complemented with *cpaL* (Fig. S5A and B). These results suggest that the increased surface adherence of the Δ*cpaL* mutant is attributable to elevated holdfast production, suggesting a role for CpaL in either the developmental or surface-sensing pathway of holdfast production.

### CpaL plays a role in surface sensing

The Δ*cpaL* HF+ mutant exhibits increased surface adhesion and holdfast synthesis in the complex medium PYE (Fig. 3A and B). In PYE, *C. crescentus* holdfast synthesis is regulated by both the developmental pathway, where holdfast is produced during differentiation from swarmer to stalked cells, and the surface-sensing pathway, where holdfast production is rapidly stimulated upon surface contact, hastening cell differentiation. In contrast, *C. crescentus* cells grown in the defined medium M2G do not undergo surface-contact-mediated holdfast synthesis (19).

To determine whether the increased surface attachment and holdfast synthesis phenotypes of the Δ*cpaL* HF+ mutant are related to the developmental or surface sensing pathway of holdfast synthesis, we performed surface attachment and holdfast quantification assays in defined M2G medium, using identical conditions as in PYE (19). First, we found that pilus production in the Δ*cpaL* HF+ mutant was comparable between M2G and PYE, with a small proportion of cells synthesizing a single long pilus, a defect that was complemented by a plasmid copy of *cpaL* (Fig. S3A and B). However, in M2G medium, the Δ*cpaL* HF+ mutant exhibited a 48% reduction in surface attachment compared to the parent HF+ strain, as expected for a mutant with reduced pilus production (Fig. 3C). Furthermore, approximately 42% of both the parental and Δ*cpaL* HF+ mutant populations produced holdfast in M2G, with no significant difference detected between these strains (Fig. 3D). These results are in stark contrast to the increased surface adhesion and holdfast synthesis phenotypes of the Δ*cpaL* HF+ mutant seen in PYE. Since surface contact does not stimulate holdfast production in M2G, these results suggest that the increased holdfast synthesis and surface attachment phenotypes of the Δ*cpaL* mutant in PYE result from the involvement of CpaL in pilus-mediated surface-sensing.

**Fig 4:**
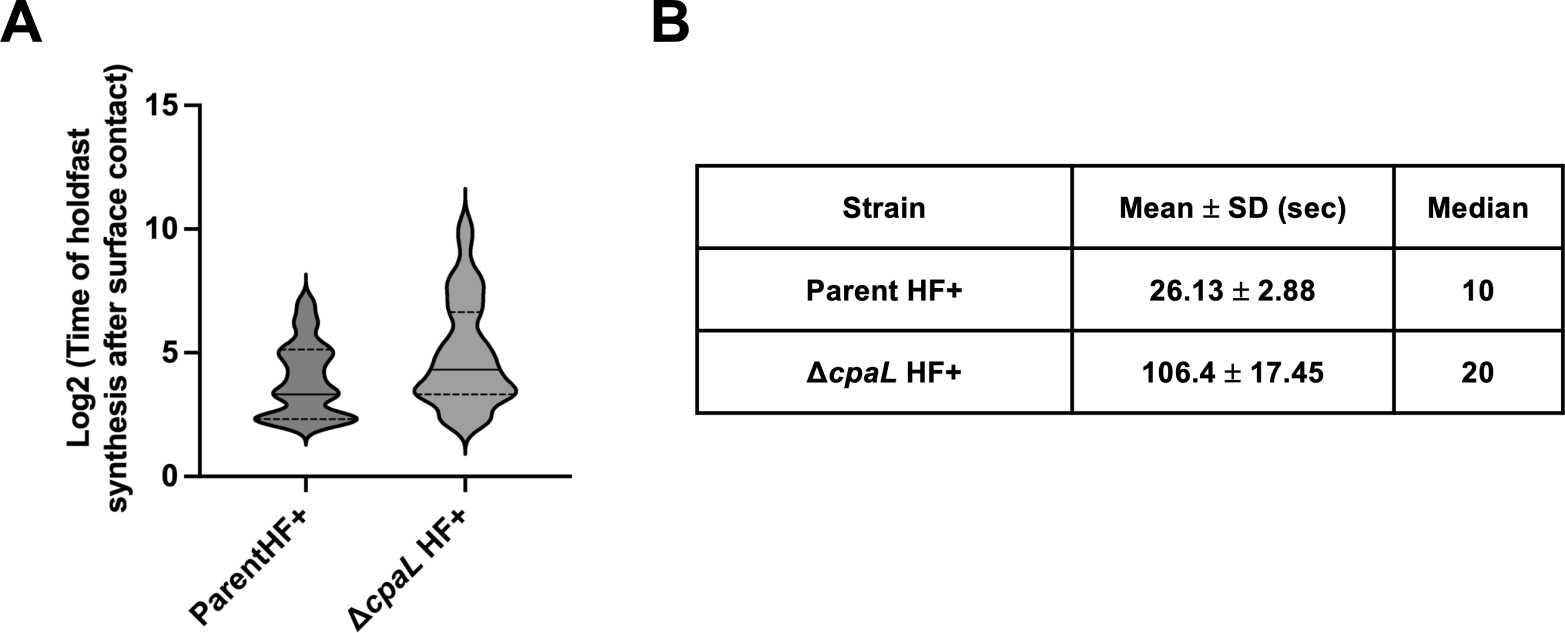
The absence of CpaL leads to a delay in holdfast production after surface contact. (**A**) Logarithmic violin plot showing the timing of holdfast synthesis after surface contact (in seconds), for three independent replicates of the parent HF+ strain (*n* = 119) and the isogenic Δ*cpaL* HF+ strain (*n* = 163). Parental background: NA1000 *pilA-cys;* HF+: *hfsA+*, holdfast positive. Statistical comparison was made using a two-tailed unpaired *t*-test. ****, *P* < 0.0001. (**B**) Table showing the means and medians of the data in the violin plot, for each of the two strains.

To further explore these findings, we examined the interaction of these cells with surfaces. Specifically, we monitored the timing of holdfast synthesis in single cells in response to surface contact in PYE medium using a polydimethylsiloxane (PDMS) microfluidic device, where cells were introduced into a well of the device and allowed to adhere to a glass cover slip in the presence of AF488-WGA. The AF488-WGA allowed us to track the formation of holdfast in individual cells upon surface contact. While the parent HF+ strain showed rapid holdfast synthesis upon surface contact, within an average of approximately 26.13 ± 2.88 s, the ∆*cpaL* HF+ mutant was comparatively slower in producing holdfast upon surface contact (106.4 ± 17.45 s; Fig. 4A and B; Movies S3 and S4). The median holdfast synthesis times for the parent HF+ strain and the Δ*cpaL* HF+ mutant were 10 and 20 s, respectively, indicating that the absence of *cpaL* renders cells less responsive to surface contact. Taken together, these results suggest that the Δ*cpaL* mutant activates holdfast production through stimulation of the surface-sensing pathway even in the absence of surfaces.

### Obstruction of pilus retraction does not increase surface attachment and holdfast production in the Δ*cpaL* mutant

**Fig 5:**
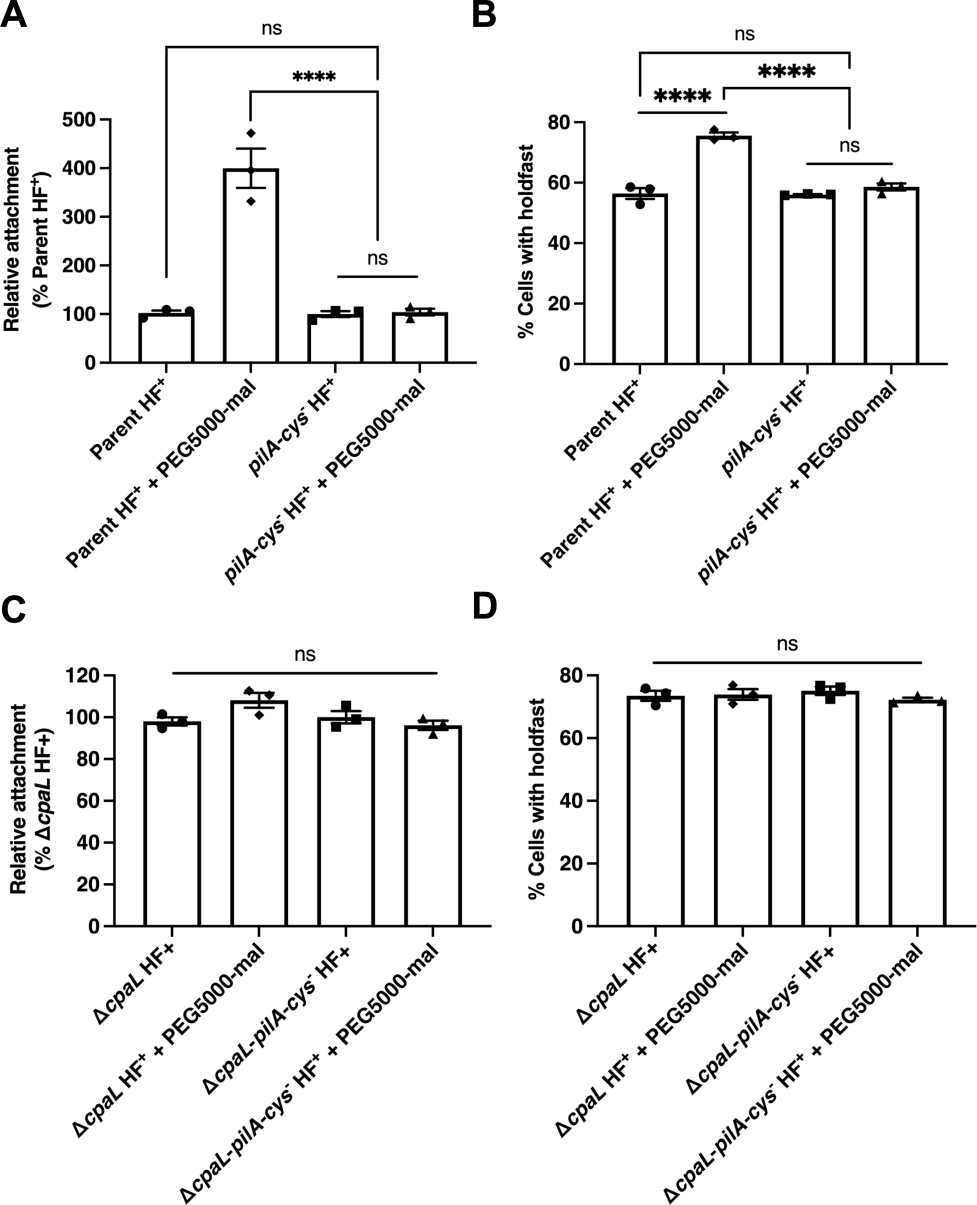
Holdfast production and attachment cannot be further stimulated by blocking pilus retraction in the ∆*cpaL* mutant. (**A**) Blocking pilus retraction with PEG5000-mal in the parent HF+ strain increases attachment. The attachment of cells to a glass coverslip after 30 min of incubation is presented relative to the parent HF+ strain in the absence of PEG5000-mal. Where indicated, PEG5000-mal was added to cultures 5 min before cells were added to the coverslip to block pilus retraction. An isogenic strain without the engineered cysteine residue in *pilA* (*pilA-cys*^-^ HF+) was used as a negative control, as pilus retraction cannot be blocked in this strain using PEG5000-mal. Parental background: NA1000 *pilA-*cys; HF+: *hfsA+*, holdfast positive. (**B**) Quantification of the percentage of cells producing holdfast in the population after 5 min of incubation with PEG5000-mal on an agarose pad. The percentage of cells producing holdfast in the population was quantified after labeling holdfast with AF488-conjugated wheat germ agglutinin (AF488-WGA). (**C**) Blocking pilus retraction with PEG5000-mal in the ∆*cpaL* HF+ strain does not increase attachment. The attachment of cells to a glass coverslip after 30 min of incubation is presented relative to the Δ*cpaL* HF+ strain (carrying the parental *pilA-cys* modification), without added PEG5000-mal. Where indicated, PEG5000-mal was added to cultures 5 min before cells were added to the coverslip to block pilus retraction. An isogenic ∆*cpaL* HF+ strain without the engineered cysteine residue in *pilA* (∆*cpaL pilA-cys*^-^ HF+) was used as a negative control, as pilus retraction cannot be blocked in this strain using PEG5000-mal. (**D**) Quantification of the percentage of cells producing holdfast in the population after 5 min of incubation with PEG5000-mal on an agarose pad, quantified after labeling holdfast with AF488-WGA. Data are the mean of three independent biological replicates. Error bars indicate the standard error of the mean. Statistical comparisons were made using Tukey’s multiple comparisons test. ****, *P* < 0.0001.

To further explore the role of *cpaL* in surface sensing, we tested whether the surface-sensing pathway could be further stimulated in the Δ*cpaL* mutant by blocking pilus retraction using PEG5000-mal. In cells carrying the *pilA-cys* modification, this treatment has been shown to stimulate the production of holdfast via the surface-sensing pathway, through steric hindrance of pilus retraction (11). Consistent with previous results, blocking pilus retraction in the parent HF+ strain containing the *pilA-cys* mutation caused a strong increase in the percentage of surface-attached cells compared to the untreated control (without PEG5000-mal), and compared to the *pilA-cys*^−^ HF+ strain lacking the *pilA*-cys modification (Fig. 5A) (11). In contrast, while the Δ*cpaL* HF+ mutant exhibited greater surface attachment compared to the parent HF+ strain under normal growth conditions (Fig. 3A), attachment of this mutant could not be further stimulated by blocking pilus retraction using PEG5000-mal (Fig. 5C).

We also compared holdfast production in these strains, finding that PEG5000-mal similarly increased holdfast production in the parent HF+ strain compared to the *pilA-cys^−^* HF+ strain, and compared to the untreated control lacking PEG5000-mal (Fig. 5B), consistent with previous results (11). However, blocking pilus retraction in the Δ*cpaL* HF+ mutant containing the *pilA-cys* modification did not further increase holdfast production compared to the untreated control or compared to the Δ*cpaL pilA-cys^−^* HF+ mutant, suggesting that the surface-sensing pathway of holdfast production was unable to further stimulate holdfast production in response to blockage of pilus retraction in the ∆*cpaL* mutant (Fig. 5D).

Collectively, these results indicate that the absence of *cpaL* leads to stimulation of the surface-sensing pathway even in the absence of mechanical cues, rendering cells less sensitive to further stimulation by blockage of pilus retraction. Thus, CpaL likely plays a role not only in pilus biogenesis but also in the regulation of the surface-sensing pathway.

### CpaL is predicted to adopt a pilin-like fold linked to a vWA domain

**Fig 6:**
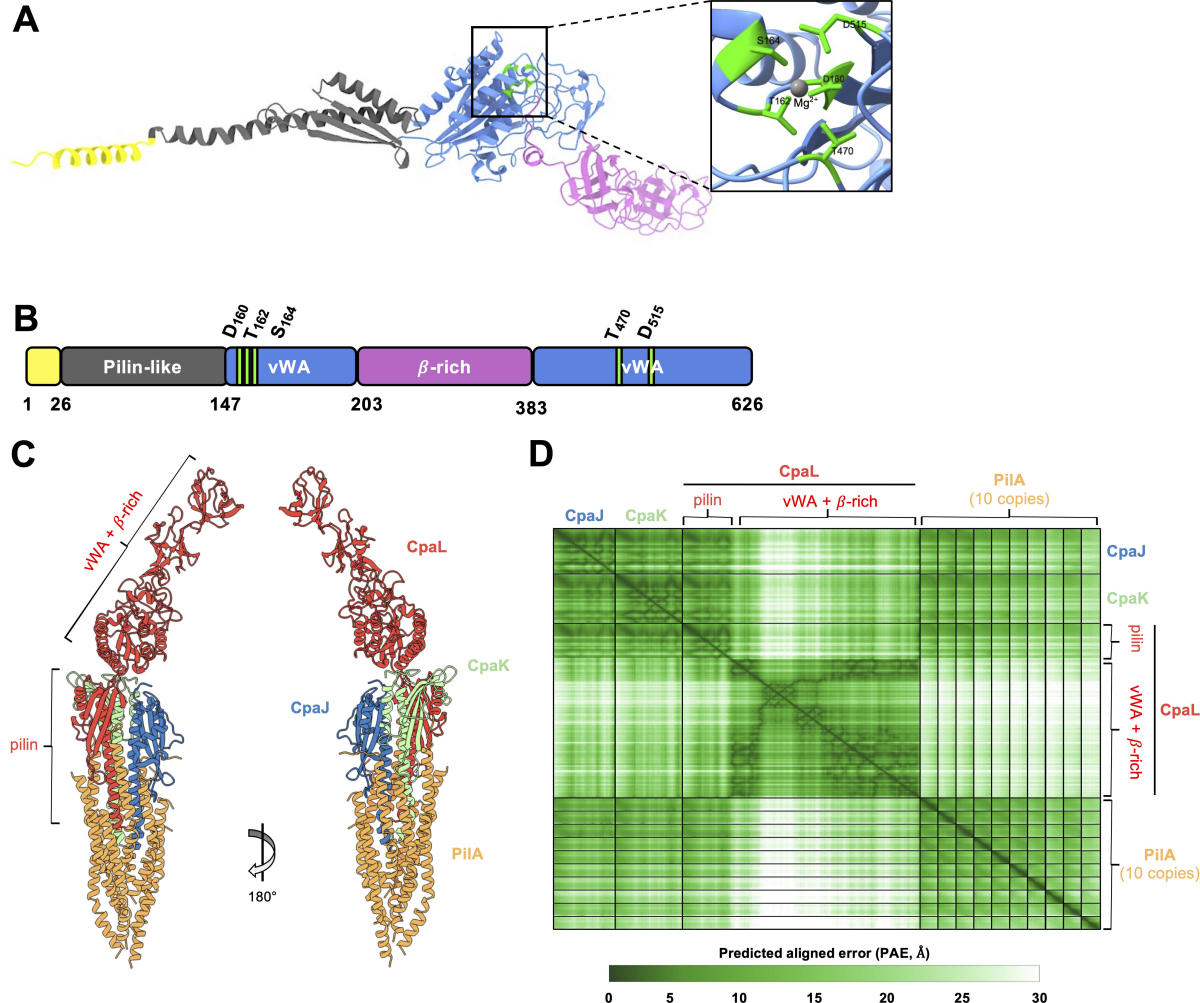
CpaL is a putative minor pilin with a vWA domain, predicted to associate with other minor pilins to form a pilus tip complex. (**A**) Ribbon diagram showing the predicted structure of CpaL generated using AlphaFold3 (pTM = 0.78). Domains and coloring are as represented in panel B. The inset depicts the metal ion-dependent adhesion site (MIDAS) motif in the vWA-like domain, which is composed of five residues (D160, T162, S164, T470, and D515, green) with a predicted Mg^2+^ ion (gray) coordinated by those residues. (**B**) Schematic representation of the CpaL domain organization. The first region at the N-terminus is predicted to be a signal peptide (residues 1–26, yellow), followed by a pilin-like module (residues 27–147, dark gray). A single vWA-like domain is divided between two distinct segments of the CpaL sequence (residues 148–203 and 384–626, blue), between which are two tandem β-rich domains (residues 204–383, pink). (**C**) Top-ranked structure of a tripartite complex composed of CpaL (red) along with the minor pilins CpaJ (blue) and CpaK (green), and 10 copies of the major pilin subunit PilA (orange) predicted by AlphaFold3 (ipTM = 0.75). The predicted or known signal sequences of each protein were removed prior to the prediction. (**D**) The predicted aligned error (PAE) scores for the model depicted in panel C. The PAE indicates the positional error in angstroms (Å) for a given pair of residues across all protein chains in the model.

To better understand the function of CpaL, we performed several sequence-based bioinformatic analyses to identify conserved domains and sequence features of CpaL. The predicted 3D structure of CpaL was generated using the AlphaFold3 server (29) (Fig. 6A; Fig. S6A and C; pTM = 0.78). The predicted structure was then submitted to the Dali server (30) to identify experimentally determined protein structures in the Protein Data Bank (PDB) that exhibit structural similarity to the predicted CpaL model (Fig. 6A). The top structural analogs of CpaL determined by Dali were all vWA-domain containing proteins that play a role in pilus or fimbrial synthesis in bacteria, including SpaC (PDB: 6M48-A) from *Lactobacillus rhamnosus*, RrgA (PDB: 2WW8-A) from *Streptococcus pneumoniae*, Mfa5 (PDB: 6TO1-A) from *Porphyromonas gingivalis*, and PilB (PDB: 7B7P-A) from *Streptococcus sanguinis*. The vWA domain is a widely distributed structural motif that, in bacteria, is characterized by its ability to mediate surface adhesion, adherence to host-derived proteins (31, 32), and mechanosensing (6) in conjunction with T4P.

The N-terminus of CpaL contains a domain of approximately 120 residues predicted to adopt a pilin-like fold (residues 27–147): a long α-helix, followed by a β-sheet consisting of three β-strands (Fig. 6A and B, dark gray), giving this module its characteristic lollipop shape (16). The predicted pilin-like module of CpaL shares structural similarity with the T4P pilin THHA1221 from *Thermus thermophilus* (PDB: 4BHR), as determined by Dali, as well as the predicted structures of the *C. crescentus* Tad minor pilins CpaK and CpaJ (Fig. S7B). Sequence analysis of this pilin-like module in CpaL revealed a potential cleavage site for the prepilin peptidase CpaA, which consists of a stretch of hydrophilic residues, followed by the consensus sequence G/A-X_4_-F/E, ending with a stretch of predominantly hydrophobic residues (16, 31) (Fig. S7A).

The C-terminus of CpaL contains the vWA-like domain, which is formed by two sections of the polypeptide chain separated by a β-rich domain (residues 148–203 and 384–626) (Fig. 6A and B, blue). This vWA domain is predicted to adopt a classical Rossmann fold that is broadly conserved across all vWA-like domains (33). Alignment of the vWA domain of CpaL with the vWA domain of the top Dali hit, SpaC of *L. rhamnosus* GG (PDB ID: 6M48), revealed significant structural similarity in the core Rossmann fold of the vWA domains of these proteins, with approximately 1 Å root mean square deviation across 118 atom pairs (Fig. S8). The vWA domain of CpaL also contains a sequence motif known as the metal ion-dependent adhesion site (MIDAS) motif (Fig. 6A and B; Fig. S8A and B), which is often present in vWA domains, where it coordinates a divalent metal ion (34). This motif is characterized by the DxS/TxS/T, T, and D signature (35), which in CpaL corresponds to D_160_, T_162_, S_164_, T_470_, and D_515_, respectively, located at the top of the central β-sheet, where they are predicted to coordinate a Mg^2+^ ion (Fig. 6A and B; Fig. S8A and B). The primary sequence that comprises the CpaL vWA domain flanks an intervening β-rich region (residues 204–384, purple) that is projected away from the MIDAS motif and contains two consecutive six-stranded β-barrels (Fig. 6A and B). A Dali search using only the structure of the β-rich region did not return any hits with strong similarity to this region of CpaL.

### CpaL is predicted to assemble at the pilus tip

The presence of a pilin-like domain in the predicted structure of CpaL (Fig. 6A; Fig. S7B) and the similarity of this structure to pilus tip-localized minor pilins suggest that CpaL may function in the context of the pilus tip. To examine this, we utilized AlphaFold3 (29) to predict a putative Tad pilus tip complex composed of CpaL, the minor pilins CpaJ and CpaK, and 10 copies of the major pilin subunit PilA to simulate a pilus filament. The sequences of the predicted or known signal sequences of each of these proteins were removed prior to the prediction. This yielded a structure in which CpaJ, CpaK, and the pilin-like domain of CpaL form a trimeric complex (Fig. 6C and D) that sits atop the 10 PilA subunits (Fig. 6C and D; Fig. S6; ipTM = 0.75), which are themselves arranged similarly to the recently resolved structure of the PilA filament (Fig. S6; PDB 8U1K) (36). The transmembrane helices of CpaJ, CpaK, and CpaL are incorporated into the distal end of the filament-like PilA structure, while the vWA domain of CpaL projects away from the rest of the complex on the opposite side (Fig. 6C and D). Importantly, predicted aligned error (PAE) scores for the model, particularly in the region comprising the interaction between CpaJ, CpaK, and the pilin-like domain of CpaL, are low (Fig. 6D), suggesting that this could represent a biological arrangement of these proteins.

### The role of CpaL in pilus-mediated mechanosensing is unique and not shared by the other minor pilins CpaJ and CpaK

**Fig 7:**
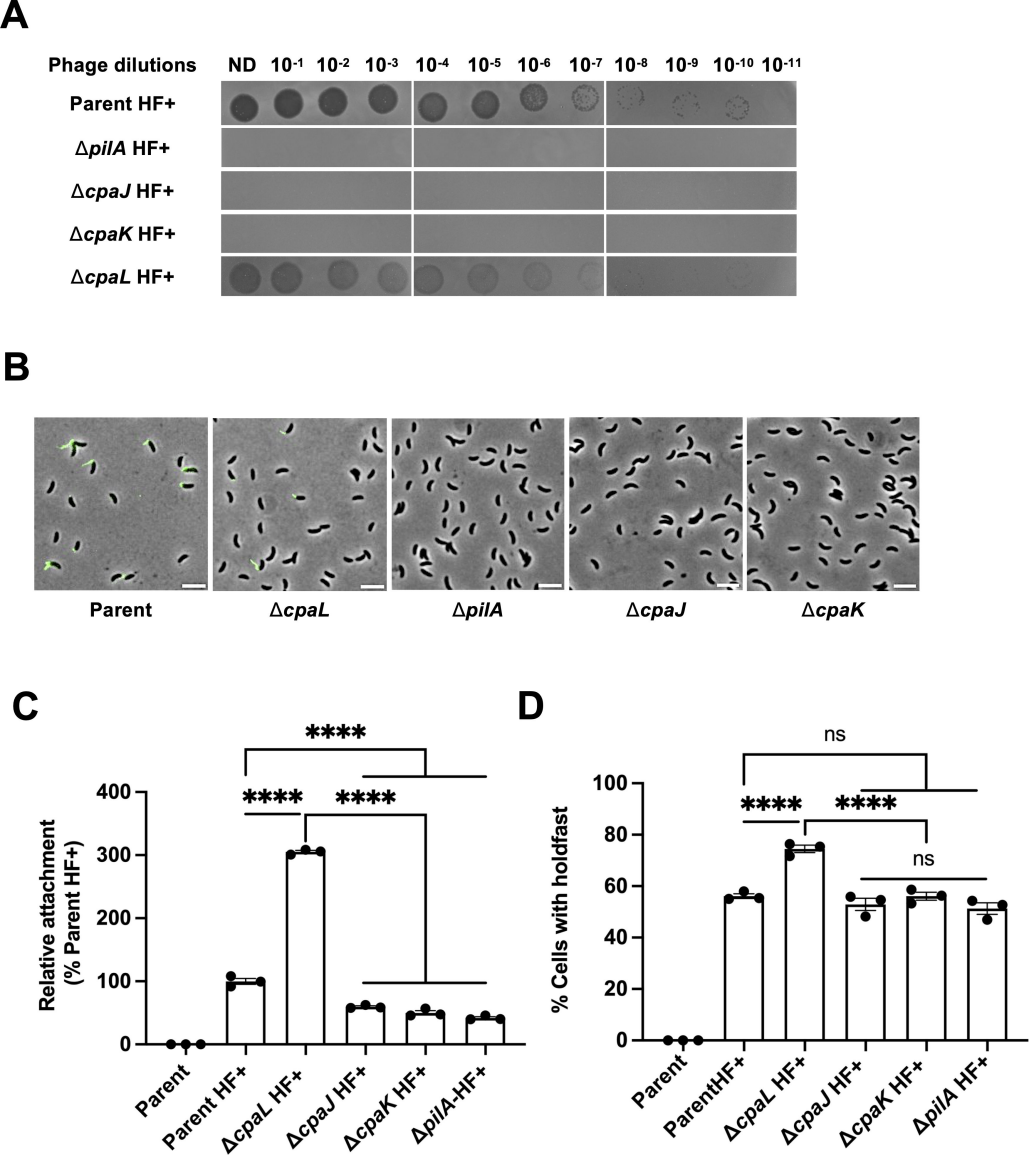
Deletion of the minor pilins *cpaJ* and *cpaK* leads to a different phenotype than deletion of *cpaL*. (**A**) Top agar phage sensitivity assays. Serial dilutions of the phage were spotted onto plates with top agar containing each bacterial strain. ND, no dilution. Parental background: NA1000 *pilA-cys*; HF+: *hfsA+*, holdfast positive. (**B**) Representative microscopy images of synchronized swarmer cells in which pilus retraction was blocked with PEG5000-mal and pili were labeled with AF488-maleimide (green). Scale bar, 10 µm. (**C**) Quantification of the attachment of cells to a glass coverslip after 30 min of incubation relative to the parent HF+ strain, grown in the complex medium PYE (Parental background: NA1000 *pilA*-cys; HF+: *hfsA+*, holdfast positive). (**D**) Quantification of the percentage of cells producing holdfast in the population in the complex medium PYE on an agarose pad. Data are the mean of three independent biological replicates. Error bars indicate the SEM. Statistical comparisons were made using Tukey’s multiple comparisons test. ****, *P* < 0.0001.

Because of the similarity of CpaL’s pilin-like domain to the minor pilins CpaJ and CpaK (Fig. S7B), and its predicted assembly with these minor pilins to form a complex at the tip of the pilus (Fig. 6C and D), we sought to determine if the minor pilins CpaJ and CpaK play a similar role in the surface-sensing pathway. We tested the effect of ∆*cpaJ* and ∆*cpaK* deletions on surface adhesion and holdfast synthesis and found that deletion of these two minor pilin genes phenocopied ∆*pilA* cells—they were resistant to the pilus-dependent phage φCbK (Fig. 7A), lacked pili (Fig. 7B), had significantly reduced surface adhesion (Fig. 7C), and produced holdfasts at levels similar to WT (Fig. 7D). Thus, we conclude that the function of CpaL is different from the functions of the minor pilins CpaJ and CpaK.

In summary, CpaL is predicted to adopt an N-terminal pilin-like fold that may allow it to incorporate into the tip of the pilus filament alongside the minor pilins CpaJ and CpaK, while its C-terminal vWA domain may play a role in mechanosensing in the pilus-mediated surface sensing pathway in *C. crescentus*.

## DISCUSSION

In this study, we demonstrate the importance of CpaL in pilus biosynthesis in *C. crescentus* and show that the absence of CpaL influences holdfast production, likely through stimulation of the surface-sensing pathway. In *C. crescentus,* the absence of pili reduces adherence to solid substrata (11). However, while deletion of *cpaL* dramatically reduces pilus synthesis (Fig. 2), this mutation leads to an unexpected increase in surface attachment and holdfast production (Fig. 3A and B). These results prompted us to explore whether CpaL may perform a role in pilus-mediated surface sensing. To examine this, we cultured *C. crescentus* in M2G minimal medium, which prevents holdfast synthesis via the surface-sensing pathway but still allows for developmentally regulated holdfast synthesis (19). Under these conditions, we found that the Δ*cpaL* mutant exhibits reduced attachment to glass, as would be expected for a mutant with impaired pilus biosynthesis (Fig. 3C), and no longer produces increased levels of holdfast (Fig. 3D). Together, these results suggest that deletion of *cpaL* stimulates holdfast biosynthesis and attachment through the surface-sensing pathway, even in the absence of surface contact. This hypothesis is supported by the absence of further stimulation of holdfast production in the Δ*cpaL* mutant upon blockage of pilus retraction with PEG5000-mal, which strongly boosts holdfast production and attachment in the parent HF+ strain (Fig. 4A and B) through activation of the surface-sensing pathway (11).

To gain insight into how CpaL may be performing seemingly opposing roles in pilus synthesis and surface sensing, we examined the predicted structure of CpaL using AlphaFold3. Analysis of its structure identified an N-terminal pilin-like module with a structural arrangement that is characteristic of pilin proteins (Fig. 6A and B; Fig. S7A and B), with particular structural homology to the major pilin THHA1221 from *T. thermophilus* (PDB: 4BHR) (37) (Fig. S7B). The pilin-like module of CpaL also shares similarity with the predicted structures of the *C. crescentus* minor pilins CpaJ and CpaK (Fig. S7B). A potential cleavage site for the *C. crescentus* pre-pilin peptidase CpaA was identified in the pilin domain of CpaL (Fig. S7A), which suggests that CpaL may be processed into a mature form to facilitate incorporation into the pilus fiber, as occurs for PilA, and potentially also for CpaJ and CpaK (Fig. S7A). These predicted features collectively suggest that CpaL could be a Tad minor pilin in *C. crescentus*. Indeed, the CpaL ortholog from the Tad pilus system of *Aggregatibacter actinomycetemcomitans*, TadG, has previously been identified as a constituent of pilus fiber preparations from this species (38–40). Similarly, future experiments should test for the presence of CpaL in the *C. crescentus* pilus fiber.

Another remarkable feature of CpaL’s domain architecture is the presence of a vWA domain, which is implicated in surface adherence and mechanosensing. Interestingly, minor pilins with vWA domains occur in several other species and have recently been structurally resolved in the T4P system of *S. sanguinis* (31, 41). These studies have demonstrated that the tripartite minor pilin complex of *S. sanguinis*, composed of PilA, PilB, and PilC, forms an “open wings” architecture that can only be accommodated at the tip of the pilus fiber (41, 42). Indeed, the incorporation of a minor pilin complex at the pilus tip is consistent with observations from the T4aP systems of *Pseudomonas aeruginosa* and *Myxococcus xanthus*. In these systems, an inner membrane complex composed of several minor pilins, as well as the vWA domain containing non-pilin subunit PilY1, recruits major pilin subunits to initiate pilus fiber assembly. This minor pilin-PilY1 complex then incorporates into the tip of the growing pilus fiber after successful nucleation of pilus assembly (43, 44). Interestingly, structural predictions with AlphaFold3 suggest that CpaL may form a tripartite complex with CpaJ and CpaK in *C. crescentus*, which may similarly localize to the distal tip of the pilus filament (Fig. 6C and D). If this complex plays a role in the initiation of pilus assembly in *C. crescentus*, it may explain the reduced piliation levels seen in the ∆*cpaL* mutantdue to infrequent nucleation of pilus assembly through disruption of the putative CpaL-CpaJ-CpaK tip complex. Indeed, infrequent nucleation of pilus assembly may also explain the longer average length of pili seen in the ∆*cpaL* mutant, as a larger pool of major pilin subunits in the inner membrane would be drawn from to assemble fewer pilus filaments (typically one per piliated ∆*cpaL* cell). Future studies should investigate the structural role of CpaL and its interactions with the other pilins to better understand its role in pilus nucleation and biosynthesis. However, our study clearly demonstrates that CpaL plays a distinct role compared to the other minor pilins in *C. crescentus*, as deletion of *cpaJ* or *cpaK* yields cells completely devoid of pili, which do not display the stimulation of holdfast synthesis and surface adhesion seen in ∆*cpaL*.

Our study also raises questions about the function of the vWA domain in CpaL (6, 33, 45) (Fig. 6A through C). In both Gram-positive and Gram-negative bacteria, T4P-associated proteins harboring vWA domains have been implicated in surface adhesion (9, 31, 32). Often, these vWA domains contain a five-residue, magnesium ion-coordinating MIDAS motif, which is required to mediate surface adherence (31). For example, the MIDAS motif in the T4P-associated protein PilC1 in *Kingella kingae* is required for adherence to host tissues, as well as for twitching motility (46). CpaL is also predicted to contain a canonical MIDAS motif with a coordinated magnesium ion (Fig. 6A and B; Fig. S11A and B), supporting CpaL’s role in surface adhesion. Additionally, it is well-documented that vWA domain-containing proteins have a mechanosensory function in eukaryotes (33). Similarly, it is thought that vWA domains could play a role in T4P-mediated mechanosensing in bacteria (6, 9). Indeed, in the pilus-tip-associated protein PilY1 in *P. aeruginosa,* the vWA domain undergoes sustained conformational changes when force is applied by AFM. This mechanosensitivity is perturbed by the mutation of a critical disulfide bond in the protein, which also reduces surface-sensing behaviors in *P. aeruginosa*, suggesting a link between force-induced conformational changes in PilY1 and surface sensing (6, 9). Given CpaL’s role in surface sensing, its predicted structure with a vWA domain, and its hypothesized localization to the pilus tip, a similar mechanism could therefore be at play in CpaL-mediated surface sensing in *C. crescentus*.

Despite the similarities of CpaL to PilB in *S. sanguinis* and PilY1 in *P. aeruginosa*, the increased surface attachment of the *C. crescentus* Δ*cpaL* mutant stands in contrast to what has been reported for the *pilB* and *pilY1* mutants, which exhibit decreased biofilm formation (9, 31), consistent with their loss of pilus production (47, 48). In fact, loss of pili in *C. crescentus* through deletion of the major pilin *pilA* also reduces biofilm formation through abolishment of pilus-mediated surface-sensing (11). However, deletion of *cpaL* does not fully abolish pilus synthesis and instead stimulates the production of holdfast via the surface-sensing pathway, similar to what is observed when pilus retraction is artificially blocked. In *C. crescentus,* it has previously been shown thatappending bulky PEG5000-mal adducts to the pilus fiber prevents pilus retraction by sterically occluding the entrance of the filament into the CpaC secretin pore in the outer membrane, thereby stimulating the surface-sensing pathway (20). Similarly, stimulation of the surface-sensing pathway was observed when a glycine to aspartate mutation was introduced in the predicted outer lip of CpaC (20), again pointing to interactions between the pilus filament and the secretin pore as key to the mechanism of surface sensing. Based on our results, we therefore hypothesize that CpaL connects with the surface-sensing pathway by modulating how the pilus fiber interfaces with CpaC and the rest of the Tad pilus secretion machinery. Evidence from *M. xanthus* and *P. aeruginosa* suggests that pilus tip proteins remain assembled as a priming complex through successive rounds of pilus extension and retraction, and that this complex acts as a plug in the secretin pore to prevent full retraction of the pilus into the inner membrane (43, 44). In these species, pilus retraction may position PilY1 within the secretin pore, where force-induced conformational changes in PilY1 due to surface contact (9) may hypothetically be sensed. By analogy, we propose that in the absence of a surface, the Tad pili in *C. crescentus* exhibit normal extension and retraction activity, without stimulating the surface-sensing pathway of holdfast synthesis. However, we further hypothesize that physical contact with a surface generates tension in the retracting pili as proposed previously (11), leading to conformational changes in CpaL. These changes may then trigger CpaC-mediated downstream signaling to stimulate the surface-sensing pathway. Notably, the absence of CpaL might mimic these tension-induced conformational changes typically induced by surface contact, effectively tricking the secretin to initiate the surface-sensing pathway for rapid holdfast synthesis even in the absence of surfaces. This suggests that CpaL may act as a mechanosensitive molecular switch translating mechanical stimuli from pilus dynamics into a developmental signaling response. Further investigation is required to better understand the exact role of CpaL in this surface-sensing mechanism and its interactions with CpaC.

In conclusion, our results identify CpaL as an important regulator of pilus-mediated holdfast biosynthesis through the surface-sensing pathway, while also implicating it in pilus synthesis. Structural predictions of CpaL reveal a pilin-like module alongside a mechanosensitive vWA domain, which may form a complex with other minor pilins to incorporate at the tip of the pilus fiber. Together, these results identify CpaL as a candidate mechanosensor for the Tad pilus in *C. crescentus*.

## MATERIALS AND METHODS

### Bacterial strains, plasmids, and growth conditions

The bacterial strains used in this study are listed in Table S2. *C. crescentus* strains were grown at 30°C in PYE (49) or in defined M2 medium supplemented with 0.2% (wt/vol) glucose (M2G) (50). PYE was supplemented with 5 µg/mL kanamycin (Kan), where appropriate. Commercial, chemically competent *Escherichia coli* DH5-α (Bioline and New England Biolabs) was used for plasmid construction and was grown at 37°C in lysogeny broth (LB) supplemented with 25 µg/mL Kan, where appropriate.

Plasmids (Table S3) were transferred to *C. crescentus* by electroporation, transduction with φCr30 phage lysates, or conjugation with S17-1 *E. coli* as described previously (51). In-frame deletion strains were made by double homologous recombination using pNPTS-derived plasmids as previously described (52). Briefly, plasmids were introduced into *C. crescentus*, and then two-step recombination was performed using kanamycin resistance to select for single crossover, followed by sucrose resistance to identify plasmid excision events. All mutants were validated by sequencing to confirm the presence of the deletion.

### Plasmid construction

The *cpaL* complementation construct was made using the high-copy-number vector pBXMCS-2 (53) with *cpaL* under the control of a xylose-inducible promoter. Because expression from this promoter is leaky, no xylose was added to the growth media for *cpaL* expression. The *cpaL* open reading frame was amplified from *C. crescentus* NA1000 genomic DNA using the cpaL-F and cpaL-R primers (Table S4). The *cpaL* PCR fragment was digested using NdeI and EcoRI and ligated into pBXMCS-2, digested by the same enzymes. Clones with a positive insert were verified by Sanger sequencing.

### Screen to identify genes involved in pilus dynamics that are not essential for pilus synthesis

To generate a transposon mutant library, 500 μL of an overnight culture of *Caulobacter pilA-cys* cells was mixed with 50 μL of exponential *E. coli* donor carrying the mariner transposon-containing plasmid pFD1 (54) and briefly vortexed. The mixture was pipetted onto a 0.22 μm filter on a vacuum manifold to concentrate cells to encourage conjugation, and the filter containing the cell concentrate was placed cell-side up on a plain PYE plate and incubated overnight at room temperature. Filter-concentrated cells (~12,000 CFUs) were resuspended in 500 μL of PYE medium and mixed with 500 μL of undiluted ~10^10^ pfu/mL φCbK phage stock (multiplicity of infection ~10^6^) and incubated at room temperature for 10 min. A total of 100 μL aliquots of cell/phage mixture were then plated on PYE plates containing kanamycin to select for transposon mutants and nalidixic acid to select against *E. coli* cells and grown at 30°C until colonies appeared (1–2 days). One hundred eighty-four colonies that were presumably φCbK phage resistant based on growth on selective kanamycin/nalidixic acid plates were inoculated into a 96-well plate containing 200 μL of PYE and grown overnight at 30°C. Cells were mixed with 20 μL of dimethyl sulfoxide and stored at −80°C for long-term storage.

Because most phage-resistant mutants derived from this selection were likely to be defective in pilus production, we sought to identify mutants that were phage-resistant but still produced pili. To identify phage-resistant, piliated mutants, we examined pilus production phenotypes for all 184 isolated colonies. Individual mutants were inoculated into 3 mL of PYE medium and grown overnight at 30°C. These overnight cultures were then labeled with 34.7 μM Alexa Fluor 488 C_5_ Maleimide (AF488-maleimide, ThermoFisher Scientific) to label pili, and incubated at room temperature for 5 min. To remove excess dye, cultures were centrifuged at 7,500 *× g* for 1 min, the supernatant was removed, and cells were resuspended in 100 μL fresh PYE before imaging. The 184 mutants fell into four general classes: (i) cells that exhibited no maleimide labeling (indicative of pilus synthesis defects), (ii) cells with aberrant growth that may or may not have exhibited maleimide labeling, (iii) cells with fluorescently labeled cell bodies but no visible pili, and (iv) cells with fluorescently labeled pilus filaments. Thirty-four mutants out of the original 184 fell into categories 2–4, exhibiting labeled T4P and/or fluorescent cell bodies indicative of T4P production and were further characterized. Irradiated φCr30 lysates of isolated transposon mutants were generated and transduced into the parent strain YB8288 to confirm that phage resistance and T4P phenotypes were dependent on transposon insertions. Twenty-five mutants with interesting phenotypes, including complete or partial phage resistance along with variable T4P fluorescence profiles, were sequenced.

To identify transposon insertion locations, genomic DNA was extracted from each strain and digested using the restriction enzyme Sau3AI at 37°C for 1 h, followed by heat inactivation at 65°C for 20 min. Digested DNA was then ligated in 100 μL reactions to promote self-annealing using T4 phage ligase at room temperature for 1 h. Ligations were then used as templates for inverse PCR reactions using primers Mariner F and Mariner R targeting the transposon element and sequenced using the same primers. Sequences adjacent to the transposon element were mapped to the *C. crescentus* genome to identify transposon insertion sites. The identities of the 25 mutants and their phenotypes can be found in Table S1.

### Phage sensitivity assays

Phage sensitivity assays were performed using the pilus-specific phage φCbK as described previously (25). Briefly, 200 μL of *C. crescentus* stationary phase culture was mixed with 3 mL of 0.5% (wt/vol) soft PYE agar. The mixture was spread over a 1.5% (wt/vol) PYE agar plate and incubated at room temperature for 1 h to solidify. A 10-fold serial dilution series of φCbK was prepared in PYE, and 5 μL of each dilution was spotted on top of the agar plate. The plates were grown for 2 days at 30°C before imaging using a ChemiDoc MP (BioRad).

### Growth rate analysis

Growth of *C. crescentus* strains was measured in 24-well polystyrene plates (Falcon) using a SpectraMax iD3 microplate reader (Molecular Devices). Stationary-phase cultures were diluted to an optical density at 600 nm (OD_600_) of 0.05, and 1 mL of this culture was added to the wells of the microplate in triplicate and incubated for 24 h at 30°C under shaking. The OD_600_ was measured at 30 min intervals to generate growth curves (OD_600_ versus time).

### Synchronization of *C. crescentus* populations

*C. crescentus* populations were synchronized to enrich for pilus-producing swarmer cells, enabling facile quantification of pilus characteristics. The swarmer cells were synchronized and collected as described previously (55) with some modifications. To do this, *C. crescentus* cultures were grown to an OD_600_ of ~0.15–0.3, and 2 mL of this culture was centrifuged at 5,400 × *g* for 5 min. The supernatant was removed, and the cell pellet was resuspended in 280 μL of PYE. Following this, 120 μL of polyvinylpyrrolidone colloidal silica solution (Percoll, Sigma) was added and mixed by gentle inversion. This mixture was centrifuged at 11,000 *× g* for 15 min, generating two bands of distinct cell populations: stalked cells in the upper band and swarmer cells in the lower band. A 15 μL aliquot of the swarmer cell band was removed and added to 85 μL of PYE. This solution of synchronized swarmer cells was washed with 100 μL of PYE before proceeding to pilus labeling, as described below.

### Pilin labeling, blocking, imaging, and quantification

The pili of *C. crescentus* were labeled as described previously (11). Briefly, 34.7 µM of AF488-maleimide was added to 100 μL of synchronized swarmer cell culture, prepared as described above, and incubated for 5 min at room temperature. Labeled cultures were centrifuged for 1 min at 5,400 *× g*, and the pellet was washed once with 100 μL of PYE to remove excess dye. The labeled cell pellets were resuspended in 7 to 10 μL of PYE, 0.5 μL of which was spotted onto a 1% agarose PYE pad (SeaKem LE, Lonza Bioscience). The agarose pad was sandwiched between glass coverslips for imaging.

To artificially block pilus retraction, methoxy-polyethylene glycol maleimide (PEG5000-maleimide, Sigma) with an average molecular weight of 5 kDa was used. 500 µM PEG5000-mal was added to 100 μL of synchronized swarmer cell cultures immediately prior to the addition of 34.7 µM AF488-maleimide. Cultures were further prepared and imaged as described above.

For observing pilus dynamics, *C. crescentus* cells were grown to an OD_600_ of ~0.15–0.3, labeled with AF488-maleimide as mentioned above, and 0.5 μL of the labeled sample was spotted onto a 1% agarose PYE or M2G medium pad as appropriate (SeaKem LE, Lonza Bioscience). The agarose pad was sandwiched between glass coverslips for imaging. Pilus dynamics were detected by time-lapse microscopy every 3 s for 1 min.

Imaging was performed using a Nikon Ti-E inverted fluorescence microscope with Plan Apo 60× or 100× objectives, a GFP filter cube, an Andor iXon3 DU885 EM CCD camera, and Nikon NIS Elements imaging software.

The number of pili per cell, the percentage of piliated cells in the whole cell population, and the percentage of cells with fluorescent cell bodies were quantified manually using ImageJ software (56).

### Surface binding assay and holdfast quantification

Surface binding assays and holdfast quantification were carried out in parallel on the same samples. For each strain, a single colony was isolated and used to inoculate 1 mL of PYE or M2G medium, as appropriate. Tenfold serial dilutions of this culture (10^−1^ to 10^−4^) were prepared and incubated overnight at 30°C. The OD_600_ of each culture was measured, and the culture with an OD_600_ closest to 0.05 was selected. All selected cultures were then normalized to exactly OD_600_ = 0.05 before proceeding with the assays to ensure consistency in cell density across samples.

For each culture prepared as described above, 15 μL was transferred onto a glass coverslip and incubated in dark and humid conditions (to prevent desiccation and effects from varying light levels) for 30 min at 30°C. After incubation, coverslips were extensively washed in water, and a 1% PYE or M2G agarose pad, as appropriate, was added on top of the cells on the coverslip for microscopy analysis.

To quantify holdfast production, cells from the same culture preparations described above (OD_600_ = 0.05) were labeled with 0.5 μg/mL AF488-WGA (Wheat germ agglutinin lectin, ThermoFisher Scientific) for 1 min before spotting them onto a 1% agarose PYE or M2G pad, as appropriate, for microscopy analysis. WGA binds specifically to the N-acetylglucosamine residues present in the holdfast polysaccharide (27).

Cells attached to the coverslip, and labeled holdfast, were imaged using a Nikon Ti-E fluorescence microscope as described above. The percentage of cells attached to the coverslip and the percentage of cells with holdfast were quantified manually from microscopy images using ImageJ software (56).

To quantify surface binding and holdfast production from cells with blocked pili, 500 µM PEG5000-mal was added to cell cultures and incubated for 5 min before performing the experiments described above.

### Timing of holdfast synthesis after surface contact

Microfluidic well devices were constructed from PDMS as described previously (11, 28). Briefly, a 10:1 mixture of PDMS prepolymer:curing agent was poured into a sterile petri plate, then the plate was placed for a few hours under a vacuum until the bubbles were removed. The plates were placed for 3 h in an oven at 65°C. A rectangle of 25 × 35 mm was cut from the PDMS plates, with fluid access holes of 3 mm in diameter punched at 5 mm intervals. Next, the glass coverslip and the PDMS cast were assembled after plasma treatment and put overnight in an oven at 65°C.

For sample preparation, 200 mL of bacterial cell culture of OD_600_ = 0.6–0.8 grown in PYE was diluted in 800 mL of PYE containing 0.5 mg/mL AF488-WGA (Wheat germ agglutinin lectin, ThermoFisher Scientific) for holdfast labeling, then 10–15 μL of the above mixture was added to one well of the PDMS device.

Time-lapse videos were taken every 5 s for 30 min at the glass-liquid interface using a Nikon Ti-E fluorescence microscope with Apo 60× objective, a GFP filter cube, an Andor iXon3 DU885 EM CCD camera, and Nikon NIS Elements imaging software. The attaching cells were detected using the phase contrast channel, while holdfast synthesis was detected using the GFP channel. The time difference between holdfast synthesis and cell-surface attachment was determined using ImageJ software (56).

### CpaL structural and functional predictions

Prediction of protein domains, their global distribution, and associated architectures was done using the Dali (30) server which was used for comparing protein structures in 3D after generation of the predicted structure of CpaL using AlphaFold3 (29). The structure of CpaL in complex with CpaJ and CpaK at the pilus tip was predicted using AlphaFold3 (29). Molecular visualization of 3D protein structures was done using the ChimeraX software (57) and AlphaFold3 (29). The GenBank accession number for the primary sequence of CpaL is WP_010918088.1, and the UniProt primary accession number is A0A0H3C404. The superposition of the vWA domain of CpaL with the vWA domain of SpaC (PDB ID: 6M48-A) was performed using ChimeraX software (57), while the superposition of the AlphaFold3-predicted PilA helical structure with the experimentally determined structure of the pilus filament (PDB 8U1K) and calculation of alignment statistics were done using MM-align (58).

### Statistical analysis

All statistical analyses were performed using GraphPad Prism 9 software. Data are presented as the mean ± standard error of the mean (SEM) for at least three biological replicates. Statistical significance was determined using a Student’s *t*-test or one-way analysis of variance, where appropriate. Further statistical details can be found in the corresponding figure legends.

## SUPPLEMENTAL MATERIAL

Legends mbio.02302-25-s0001.docx Legends for Movies S1 to S4.

Supplemental material mbio.02302-25-s0002.pdf Fig. S1 to S8; Tables S1 to S4.

Movie S1 mbio.02302-25-s0003.mp4 Non-synchronized cells of the parent strain (NA1000 *pilA-cys*) showing the extension-retraction dynamics of pili labeled with AF488-maleimide.

Movie S2 mbio.02302-25-s0004.mp4 Non-synchronized ∆*cpaL* cells in the parental background (NA1000 *pilA-cys*) showing the extension-retraction dynamics of pili labeled with AF488-maleimide.

Movie S3 mbio.02302-25-s0005.avi Cells from the parent HF+ strain producing holdfast upon surface contact.

Movie S4 mbio.02302-25-s0006.avi ∆*cpaL* HF+ cells producing holdfast upon surface contact in the presence of AF488-WGA.

ASM does not own the copyrights to Supplemental Material that may be linked to, or accessed through, an article. The authors have granted ASM a non-exclusive, world-wide license to publish the Supplemental Material files. Please contact the corresponding author directly for reuse.
