# Supplementary material for: Stimulation of the Caulobacter crescentus surface sensing pathway by deletion of a specialized minor pilin-like gene
Source: mBio. 2025 Oct 1;16(11):e02302-25. doi: 10.1128/mbio.02302-25 (PMC12607857; doi:10.1128/mbio.02302-25)
Supplement: Legends — for Movies S1 to S4. [file mbio.02302-25-s0001.docx]

Movie S1. Time-lapse movie of non-synchronized cells of the parent strain (NA1000 pilA-cys) showing the extension-retraction dynamics of pili labeled with AF488-maleimide (green). Frame rate is 5 fps. Scale bar, 5 μm.

Movie S2. Time-lapse movies of non-synchronized ∆cpaL cells in the parental background (NA1000 pilA-cys) showing the extension-retraction dynamics of pili labeled with AF488-maleimide (green). Frame rate is 5 fps. Scale bar, 5 μm.

Movie S3. A representative time-lapse movie of cells from the Parent HF + strain producing holdfast upon surface contact. Holdfast was labeled with AF488-WGA. Parental background: NA1000 pilA-cys; HF+: hfsA+, holdfast positive. The cell bodies are in gray and the holdfasts are in green. Frame rate is 25 fps. Scale bar, 5 μm.

Movie S4. A representative time-lapse movie of ∆cpaL HF+ cells producing holdfast upon surface contact in the presence of AF488-WGA. The cell bodies are in gray and the holdfasts are in green. Frame rate is 25 fps. Scale Bar, 5 μm.
