# Supplementary material for: Stimulation of the Caulobacter crescentus surface sensing pathway by deletion of a specialized minor pilin-like gene
Source: mBio. 2025 Oct 1;16(11):e02302-25. doi: 10.1128/mbio.02302-25 (PMC12607857; doi:10.1128/mbio.02302-25)
Supplement: Supplemental material — Fig. S1 to S8; Tables S1 to S4. [file mbio.02302-25-s0002.pdf]

Figure S1

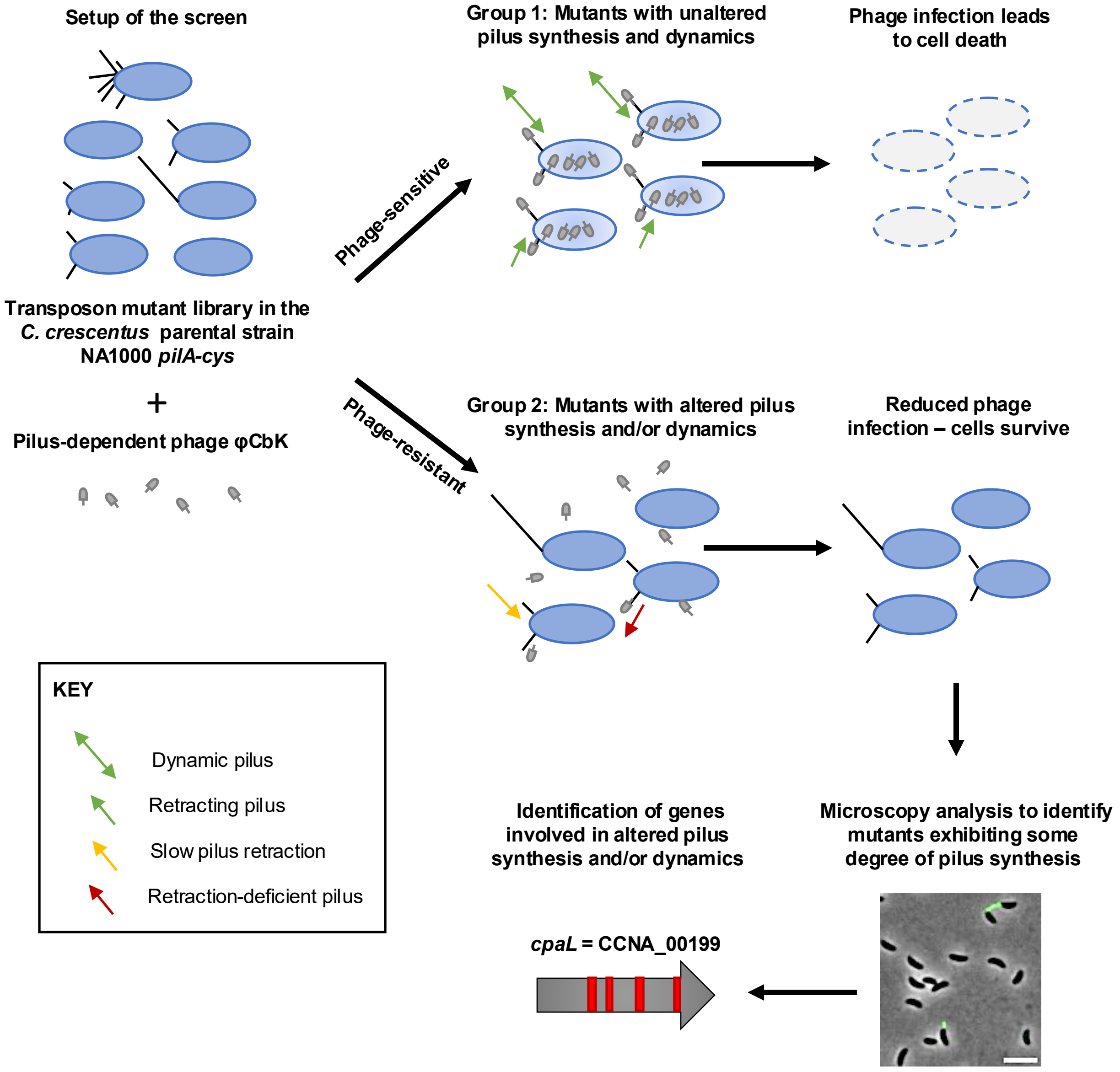

Figure S1: Genetic screen for resistance to a pilus-dependent phage identifies *cpaL* as a mediator of pilus synthesis and/or dynamics. Schematic of the forward genetic screen used to identify mutants involved in pilus dynamics. A *C. crescentus Mariner* transposon mutant library was generated in the parent strain (NA1000 *pilA*-cys), and mixed with the pilus-dependent phage ϕCbK and grown on plates. Mutants with complete or partial resistance to phage were isolated and imaged for pilus synthesis (See Methods). Red bars indicate the position of transposon insertions identified within the *cpaL* (CCNA_00199) gene coding sequence (gray arrow). Scale bar, 10 µm.

Figure S2

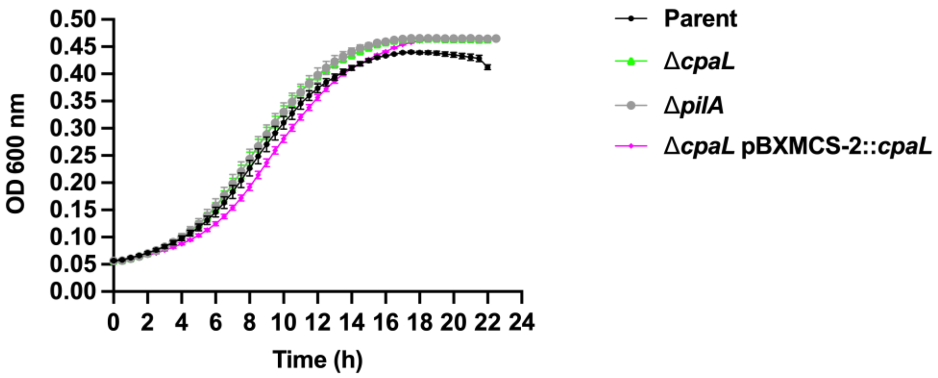

Figure S2: **The growth rate of *C. crescentus* is not affected by the absence of *cpaL*.**

Growth curves of the indicated strains of *C. crescentus* grown in PYE. OD_{600} was measured every 30 min and is the average of six independent biological replicates. The parent (NA1000 *pilA-cys*), Δ*pilA*, and Δ*cpaL* strains contain the empty vector plasmid. Error bars represent the standard error of the mean.

Figure S3

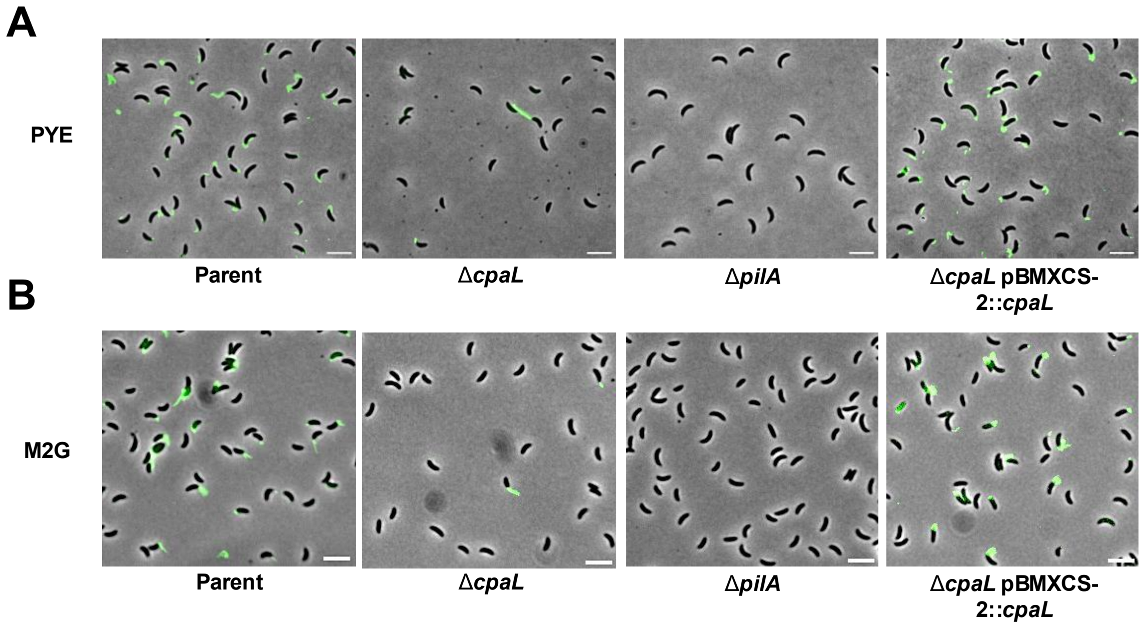

Figure S3: Representative microscopy images showing piliation in the key strains used in this study.

Synchronized swarmer cells grown in **A**: complex PYE medium, or **B**: minimal M2G medium, were blocked for pilus retraction with PEG5000-mal and labeled with AF488-maleimide (green). The parent (NA1000 *pilA-cys*), Δ*cpaL*, and Δ*pilA* strains carry the empty plasmid vector, whereas the Δ*cpaL* complementation strain carries the pBMXCS-2::*cpaL* plasmid. Scale Bar, 10 µm.

Figure S4

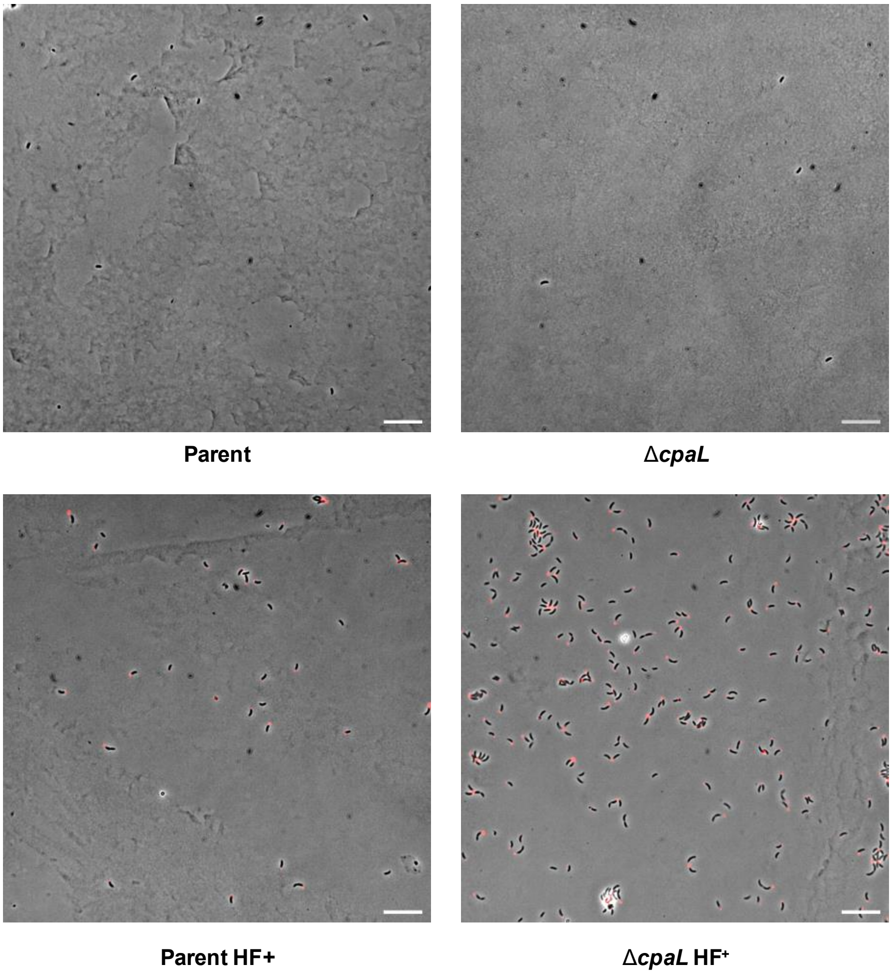

Figure S4: Representative microscopy images for the attached cells to the surface.

Cells are grown in the complex medium PYE and incubated for 30 min to a glass coverslip (Parent background: NA1000 *pilA*-cys; HF+: *hsfA*$^{+}$, Holdfast positive). Holdfasts are labeled with the AF488 conjugated wheat germ agglutinin (AF488-WGA). Scale Bar, 10 μm.

Figure S5

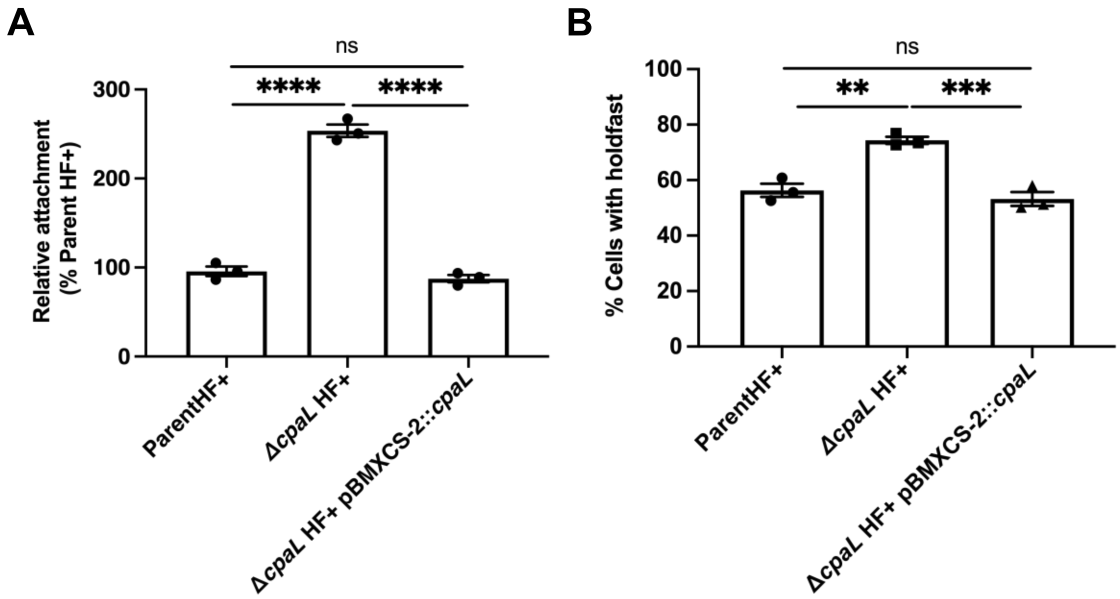

Figure S5: Plasmid complementation of the Δ*cpaL* mutant restores attachment and holdfast production to wild-type levels.

A: Quantification of the attachment of cells to a glass coverslip after 30 min of incubation, when grown in the complex medium PYE. Attachment values are presented relative to the Parent HF+ strain. Parental background: NA1000 *pilA-cys*; HF+: *hfsA*+, holdfast positive. Isogenic Δ*cpaL* and ΔcpaL pBMXCS-2::*cpaL* strains carrying the HF+ modification are presented. **B:** Quantification of the percentage of cells producing holdfast in the population, when grown in the complex medium PYE on an agarose pad. In panels A and B, the Parent HF+ and Δ*cpaL* HF+ strains carry the empty plasmid vector. Data are the mean of three independent biological replicates. Error bars indicate the standard error of the mean. Statistical comparisons were made using Tukey's multiple comparisons test. ****, P <0.0001; ***, P <0.001; **, P <0.01.

Figure S6

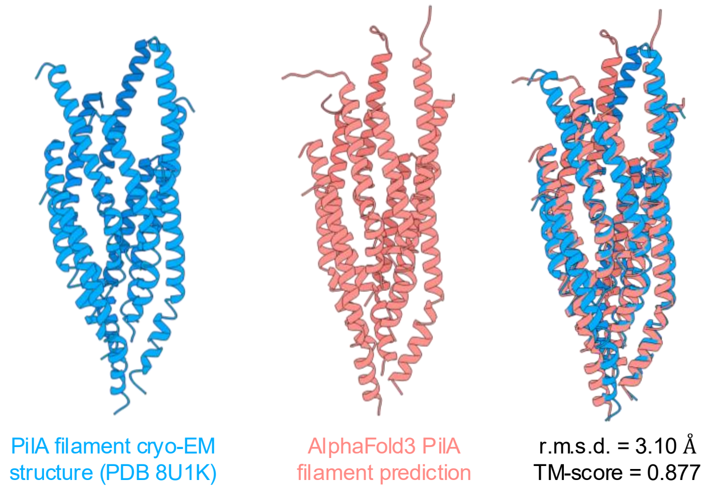

Figure S6: Alignment of the structure of the *C. crescentus* PilA filament determined by cryo-EM (from PDB 8U1K) and the structure of the PilA filament as determined by AlphaFold3 (from the prediction in the figure 6C).

Figure S7

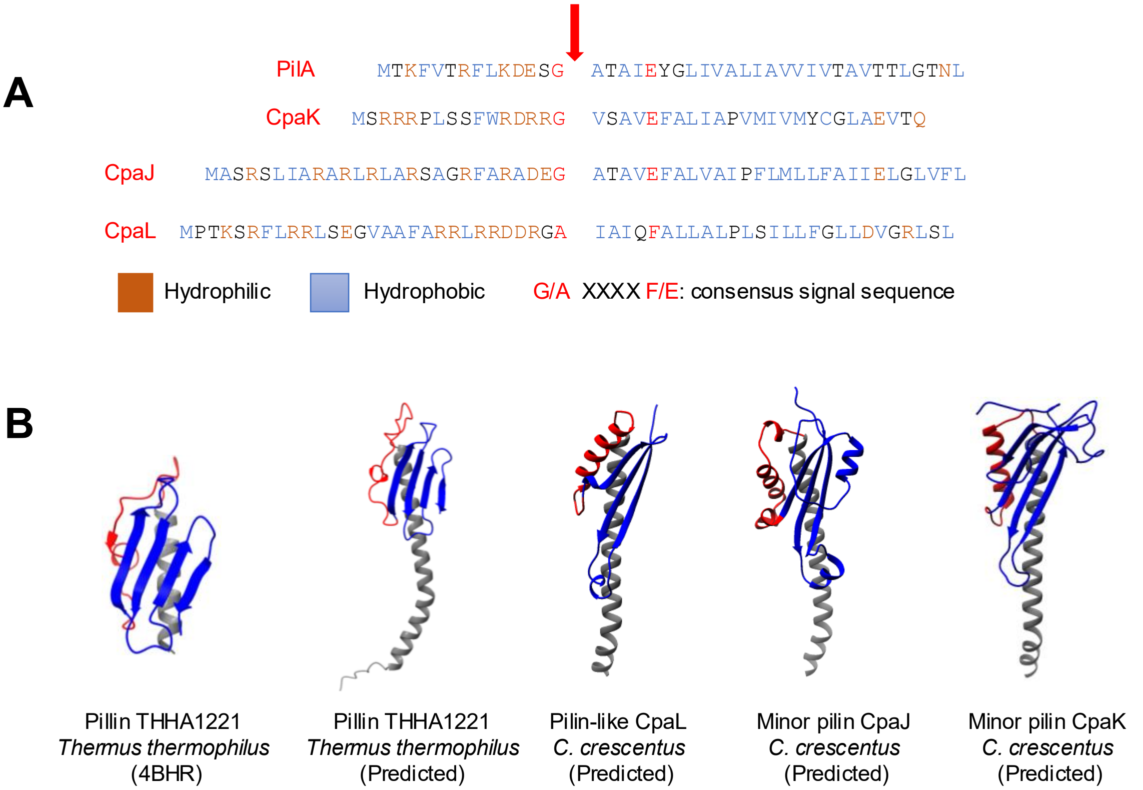

Figure S7: CpaL has a pilin-like module with a predicted prepilin peptidase (CpaA) cleavage site.
A: The N-terminal sequences of PilA, minor pilins CpaK and CpaJ, and CpaL. Hydrophilic residues are highlighted in orange, and hydrophobic residues are highlighted in blue. The consensus sequence (G/A-X-X-X-F/E) for recognition by the prepilin peptidase CpaA is shown by highlighting the G/A and F/E residues in red. The predicted CpaA cleavage site is indicated with a red arrow. **B:** Structural comparison of the experimentally determined and AlphaFold3-predicted structures of the T4P THHA1221 from Thermus thermophilus (PDB:4BHR), along with the predicted pilin-like modules of CpaL, CpaJ, and CpaK, with their predicted signal sequences removed.

Figure S8

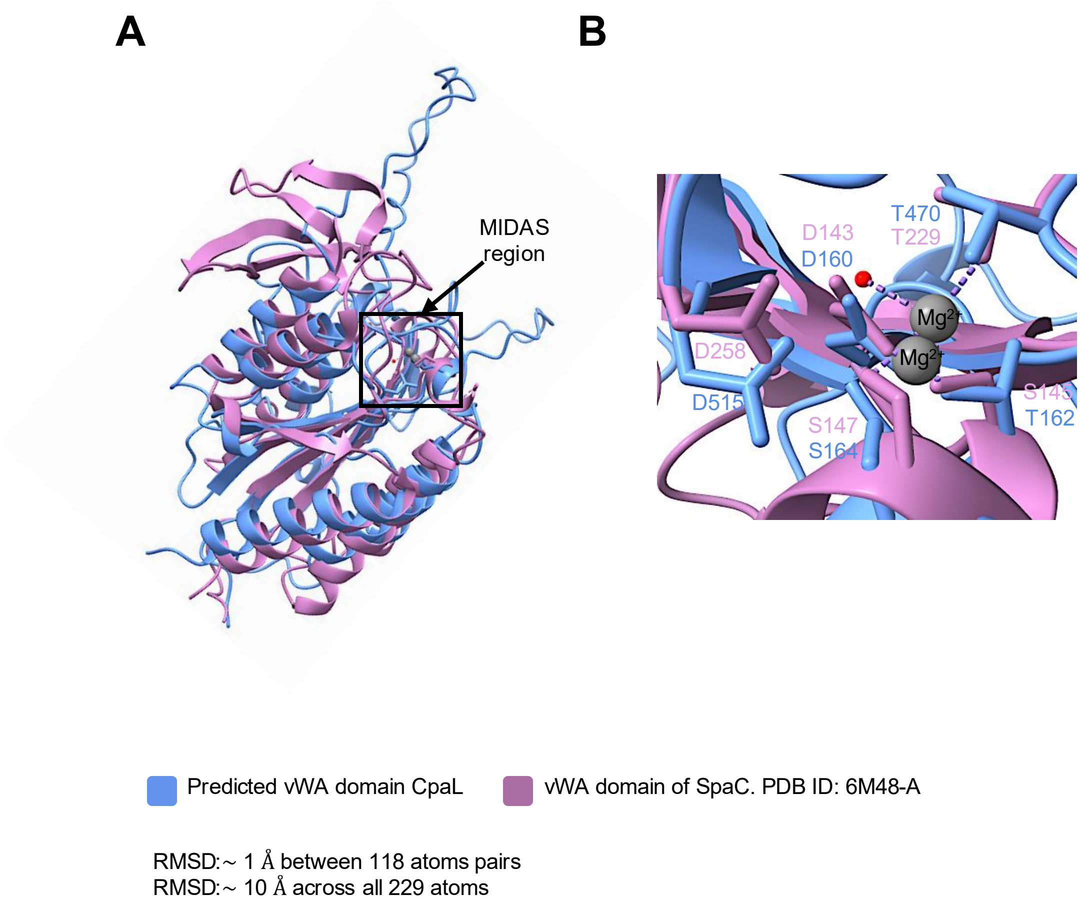

Figure S8: Structural comparison of the vWA domains of CpaL and the SpaC protein of *Lactobacillus rhamnosus* GG.

A: Structural superposition of the predicted vWA domain of CpaL (blue, residues 148-203, and 383-626) with the vWA domain of SpaC (pink, residues 129-386) determined by X-ray crystallography (PDB ID: 6M48-A). **B:** Magnified image of the box in panel A depicting the MIDAS motif regions of CpaL (blue) and SpaC (pink). The Mg^{2+} ion that co-crystallized with SpaC, as well as the Mg^{2+} ion predicted by AlphaFold3 in the vWA domain of CpaL, are represented by two large grey spheres, while the water molecule involved in Mg^{2+} ion coordination by SpaC is depicted as a small red sphere. RMSD: ~ 1 Å between 118 atom pairs; RMSD: ~ 10 Å across all 229 atoms.

<u>Tables:</u>

Table S1:

**Mutant name**	**Microscopy phenotype of transduced strain**	**Qualitative phage sensitivity phenotype**	**Gene insertion**	**Putative function**
P1A3	labeled pili	some φCbK resistance	CCNA_00199	VWA pilus gene (*cpaL*)
P1A6	division defect, some labeled pili	some φCbK resistance	CCNA_03136	class II flagellar gene (*fliJ*)
P1B2	fluorescent cell bodies	some φCbK resistance	CCNA_00199	VWA pilus gene (*cpaL*)
P1B6	labeled pili	some φCbK resistance	CCNA_01525	flbT-flagellar biosynthesis repressor
P1C5	labeled pili	some φCbK resistance	CCNA_00199	VWA pilus gene (*cpaL*)
P1C8	fluorescent cell bodies	φCbK resistant	CCNA_03044	*cpaC*-related pilus protein
P1D9	labeled pili	some φCbK resistance	CCNA_00942	*flgL*-flagellar hook gene
P1E1	some labeled pili	some φCbK resistance	CCNA_03280	pyruvate ferredoxin/flavodoxin

				oxidoreductase family protein
P1E2	fluorescent cell bodies	φCbK resistant	CCNA_00199	VWA pilus gene (*cpaL*)
P1E3	fluorescent cell bodies	φCbK sensitive	CCNA_00653	*nasC*-nitrate reductase
P1E5	fluorescent cell bodies	some φCbK resistance	CCNA_02961	*neuB*-family N-acetylneuraminate synthase
P1E6	no labeled pili	φCbK resistant	CCNA_02567	*pleC*
P1F2	labeled pili	some φCbK resistance	CCNA_00950	flagellar protein *fliF*
P1F3	labeled pili	φCbK sensitive	CCNA_02661	M16 family peptidase
P1G2	fluorescent cell bodies	some φCbK resistance	CCNA_02742	hypothetical protein
P1H2	aberrant growth	some φCbK resistance	CCNA_02742	hypothetical protein
P1H9	aberrant growth	φCbK resistant	CCNA_02567	*pleC*
P1H10	labeled pili	some φCbK resistance	CCNA_03578	*recQ*
P1H12	aberrant growth	φCbK resistant	CCNA_02567	*pleC*

P2B2	labeled pili	some φCbK resistance	CCNA_00956	*flhA*
P2C2	labeled pili	some φCbK resistance	CCNA_03404	*rcdA*
P2C3	labeled pili	some φCbK resistance	CCNA_00747	*tipF*-flagellar regulator
P2G3	labeled pili	some φCbK resistance	CCNA_00638	heme binding protein
P2H3	labeled pili	some φCbK resistance	CCNA_02153	acetylspermidine deacetylase

Table S2: Strains used in this study

**Strain designation**	**Genotype and method of construction**	**Source or reference**
Caulobacter crescentus		
YB8220	NA1000 *hfsA*+ *pilA*^{T36C}	(11)
YB6374	NA1000 *hfsA*+ Δ*pilA*	(11)
YB8644	NA1000 *hfsA*+ *pilA*^{T36C} Δ*cpaL* (Transduced strain YB8215 with lysate made from strain YB8287)	This study
YB8454	NA1000 *pilA*^{T36C} Δ*cpaK* (Electroporated plasmid pNPTS138ΔcpaK from strain 8453 into strain 8288.	This study

YB10278	NA1000 *pilA*^{T36C} Δ*cpaJ* (Electroporated plasmid pNPTS138ΔcpaJ from strain 10277 into strain 8288.	
FC764	NA1000 *hsfA*+	(26)
YB8215	NA1000 *hfsA*+ Δ*cpaL* (electroporated plasmid from strain YB8206 into strain FC764)	This study
YB8288	NA1000 *pilA*^{T36C}	(11)
LS3118	NA1000 Δ*pilA*	(22)
YB8294	NA1000 *pilA*^{T36C} Δ*cpaL* (Electroporated strain YB8288 with plasmid from strain Y8206)	This study
YB9770	NA1000 *pilA*^{T36C} pBXMCS-2 (Electroporated strain YB8288 with plasmid pBXMCS-2)	This study
YB9771	NA1000 Δ*pilA* pBXMCS-2 (Electroporated strain LS3118 with plasmid pBXMCS-2)	This study
YB9772	NA1000 *pilA*^{T36C} Δ*cpaL* pBXMCS-2 (Electroporated strain YB8294 with plasmid pBXMCS-2)	This study

YB9742	NA1000 *pilA*^{T36C} Δ*cpaL* pBXMCS-2 *cpaL* (YB9741 Electroporated strain YB8294 with plasmid from YB9741)	This study
YB9773	NA1000 *hfsA*+ *pilA*^{T36C} pBXMCS-2 (Electroporated strain YB8220 with plasmid pBXMCS-2)	This study
YB9774	NA1000 *hfsA*+ *pilA*^{T36C} Δ*cpaL* pBXMCS-2 (Electroporated strain YB8644 with plasmid pBXMCS-2)	This study
YB9775	NA1000 *hfsA*+ Δ*pilA* pBXMCS-2 (Electroporated strain YB6374 with plasmid pBXMCS-2)	This study
YB9776	NA1000 *hfsA*+ *pilA*^{T36C} Δ*cpaL* pBXMCS-2 *cpaL* (Plasmid from YB9741 electroporated into strain YB8644 by electroporation)	This study
YB9777	NA1000 *hfsA+ pilA*^{T36C} *ΔcpaJ* (Electroporated strain YB 8220 with plasmid pNPTS138 Δ*cpaJ* from YB10277)	This study
YB9778	NA1000 *hfsA+ pilA*^{T36C} *ΔcpaK* (Electroporated strain YB 8220 with	This study

	plasmid pNPTS138 Δ*cpaK* from YB 8453)	
Escherichia coli		
YB4030	S17-1/pNPTS138 Δ*pilA*	(19)
YB8206	α-select/pNPTS139 Δ*cpaL*	This study
YB8286	α-Select/pNPTS139 *pilA*^{T36C}	(11)
YB8453	α-select/pNPTS138 Δ*cpak*	This study
YB102777	α-select/pNPTS138 Δ*cpaJ*	This study
YB9741	NEB 5-alpha/pBXMCS-2 *cpaL*	This study

Table S3: Plasmids used in this study

**Plasmid**	**Description**	**Source or reference**
pNPTS138	Litmus 38 derivative, *oriT sacB* Kan^r	M. R. K. Alley
pNPTS139	Litmus 39 derivative, *oriT sacB* Kan^r	M. R. K. Alley
pNPTS139 *pilA*^{T36C}	pNPTS139 containing 500-bp fragments upstream and downstream of *pilA*^{T36C} point mutation	(11)
pNPTS138 Δ*pilA*	pNPTS138 containing 480 bp fragments upstream and downstream of *pilA*	(19)
pNPTS139 Δ*cpaL*	pNPTS139 containing 500-bp fragments upstream and downstream of *cpaL*	This study
pNPTS138 Δ*cpaJ*	pNPTS138 containing 500 bp fragments upstream and downstream of *cpaJ*	This study
pNPTS138 Δ*cpaK*	pNPTS138 containing 500 bp fragments upstream and downstream of *cpaK*	This study
pBXMCS-2	High copy replicating plasmid, xylose inducible promotor, Kan^r	(53)
pBXMCS-2 *cpaL*	pBXMCS-2 containing *cpaL* open reading frame	This study

Table S4: Primers used in this study

Primer name	Sequence*	Function
cpaL-UP F	**TTC TGG ATC CAC GAT** CGA GCA CCC GCT GGT CCA	pNPTS139Δ*cpaL* construction
cpaL-UP R	TCT CAG ACG GCG GGC GAA A	
cpaL-DN F	TCT TTC CAG GCC ATT GCT C	
cpaL-DN R	**AGC TTC CTG CAG GAT** GCA GGA CGG CAA TTC GGT	
cpaL-F	GCA **CAT ATG** ATG CCG ACC AAG AGC CGT TT	pBXMCS-2 *cpaL* construction
cpaL-R	GGG **GAA TTC** CTA TCT GGC GAT CCG CAG GT	
cpaJ-UP F	**GCC AAG CTT CTC TGC AGG AT**T CGA GCC GGT TTC CGC GAA G	pNPTS138Δ*cpaJ* construction
cpaJ-UP R	*GGT GAA CGA GGT GGC GGC GTA* GAT GAG GGA GCG TGA AGC CA	
cpaJ-DN F	TAC GCC GCC ACC TCG TTC ACC	
cpaJ-DN R	**GCG AAT TCG TGG ATC CAG AT**G AGG TGT AGA CAT AGC TCG CC	
cpaK-UP F	**GC GAA TTC TGG ATC CAC GAT** CAC GTC TCC GGC TGG CGC GTT	pNPTS138Δ*cpaK* construction
cpaK-UP R	*T CTC GGC CTT* GCG CCA GAA GCT GGA AAG GGG GCG	
cpaK-DN F	**C TTC TGG CGC** AAG GCC GAG ACG ATC CTC TGG TCG ACG	

cpaK-DN R	**CA GAA AGC TTC CTG CAG GAT** GGT CGC GGG CTT GAT CGG CCC	
Mariner F	ACG GTA TCG ATA AGC TTG ATA TCG A	
Mariner R	CAG AGT TGT TTC TGA AAC ATG GCA	

*Restriction sites and regions of plasmid complementarity to facilitate Gibson assembly are in bold; regions of complementary to the target amplicon are underlined; regions of reverse complementarity (to facilitate allele assembly) are italicized.
